# Supplementary material for: Catalytic carbon–carbon bond cleavage in lignin via manganese–zirconium-mediated autoxidation
Source: Nat Commun. 2024 Jan 29;15:862. doi: 10.1038/s41467-024-45038-z (PMC10825196; doi:10.1038/s41467-024-45038-z)
Supplement: Supplementary file 1 — Supplementary Information [file 41467_2024_45038_MOESM1_ESM.pdf]

Supplementary Information

Catalytic carbon–carbon bond cleavage in lignin via manganese–zirconium-mediated autoxidation

Chad T. Palumbo,[1] Nina X. Gu,[1] Alissa C. Bleem,[1] Kevin P. Sullivan,[1] Rui Katahira,[1] Lisa M. Stanley,[1] Jacob K. Kenny,[1,2] Morgan A. Ingraham,[1] Kelsey J. Ramirez,[1] Stefan J. Haugen,[1] Caroline R. Amendola,[1] Shannon S. Stahl,[3,*] and Gregg T. Beckham[1,*]

1. Renewable Resources and Enabling Sciences Center, National Renewable Energy Laboratory, Golden, Colorado 80401, United States.
2. Department of Chemical and Biological Engineering, University of Colorado Boulder, Boulder 80303, CO, United States.
3. Department of Chemistry, University of Wisconsin-Madison, Madison, Wisconsin 53706, United States. Great Lakes Bioenergy Research Center, University of Wisconsin-Madison, Madison, Wisconsin 53706, United States.

Supplementary Table of Contents

Supplementary Methods 2
- Chemicals and biomass feedstocks 2
- Analytical methods 2
- Synthesis, Oligomer Substrate Preparation, and Autoxidation Catalysis 6
- Model Compound and Analyte Synthesis 6
- Autoxidation of the Model Compounds 18
- Oligomer Substrate Preparation 19
- Autoxidation Catalysis of the Oligomer Fractions 23
- Strain construction 26
- Preparation of bacterial culture media 26
- Shake flask cultivations 26
- Plasmids 26
- DNA Sequences 27
- Synthetic Genes 28
- Strains and Construction Details for Bacterial Strains 29
- Initial Compositions of Bacterial Growth Media 30
- Numerical data for metabolites measured during cultivation of ACB263 30
- Bioconversion Conversion 32

Supplementary References 34

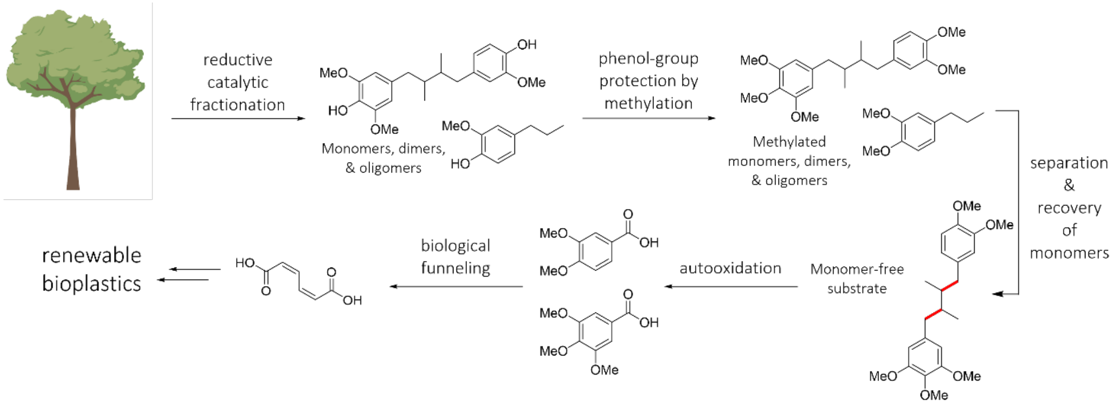

Supplementary Fig. 1. Scheme illustrating the overall process for converting biomass to *cis,cis*-muconic acid. Created with BioRender.com

Supplementary Methods

Chemicals and biomass feedstocks

Unless otherwise stated, all reagents were purchased from commercial sources and used without further purification. The compounds 4-propenylsyringol,[1, 2] 2,2',3,3'-tetramethoxy-5,5'-dipropyl-1,1'-biphenyl,[3, 4, 5] **6**, 2,2',3,3'-tetramethoxy-1,1'-dicarboxylic acid-5,5'-biphenyl,[6] 2-hydroxypropioveratrone,[7] dimethyl 2-methoxymaleate,[8] Dimethyl 5,5',6,6'-tetramethoxy-biphenyl-3,3'-dicarboxylate,[9] and 2-acetoxy-1,3-dimethoxy-5-propyl-benzene[10] were prepared according to the literature.

Clean chips of hybrid poplar clone OP-367 (*P. deltoides x P. nigra)* harvested by Greenwood Resources in 2013 from Morrow County, OR were used. They were further refined in a bliss hammermill to pass through a ¼" sieve. The final milled particles were sieved through a 2 mm screen. Loblolly pine was used that was obtained by FTX Consulting from Edgefield South Carolina in 2018 and passed through a ¼" screen prior to use. The biomass was extracted according to a modified version of the NREL laboratory analytical procedure[11, 12] for Soxhlet extraction of biomass. To accommodate a larger sample, the extraction was performed at an increased scale of 800 g of biomass in a custom made Soxhlet apparatus.

Analytical methods

GC-FID quantification of RCF monomers and autoxidation products

GC-FID was performed on an Agilent Technologies 8890 autosampler. GC-MS was performed on an Agilent Technologies 7890A autosampler equipped with a 5975C inert XL MSD with a Triple-Axis Detector. The GC-MS was operated with the same temperatures, injection volumes, and programmed temperature ramps as those used for GC-FID (see below) to enable comparable retention times. ^{1}H (500 MHz) and ^{13}C (125 MHz) NMR spectroscopy was performed on Bruker 400 MHz and 300 MHz NMR spectrometers. A 1 μL injection was used with a split ratio of 10:1. A 30 m x 250 μm x 0.25 μm Agilent Technologies HP-5ms column was used. The inlet temperature was set to 260 °C. The oven was initially held at 50 °C for 2 min and then ramped 40 °C min^{-1} until 100 °C. The ramp was then changed to 5 °C min^{-1} until 110 °C where it was held for 5 min. The method continues to ramp at 5 °C min^{-1} until 160 °C where temperature is held for 2 min. The temperature is then ramped at 25 °C min^{-1} until 300 °C where it is held for 2 min, reaching a total duration of 29.85 min. A flame ionization detector was used to quantify the products, and calibrations curves were generated for the oxidation products based on peak area ratios between analyte standards and a naphthalene internal standard. For the mixed aldehyde ester biphenyl product, 2,2',3,3'-tetramethoxybiphenyl-1'-formyl-1-carboxylic acid, a calibration curve was generated by averaging the peak areas of dimethyl divanillin and tetramethyl divanillate, as shown in Supplementary Fig. 2 and Supplementary Tables 1-2. GC-MS was performed on an Agilent Technologies 7890A autosampler equipped with a 5975C inert XL MSD with a Triple-Axis Detector.

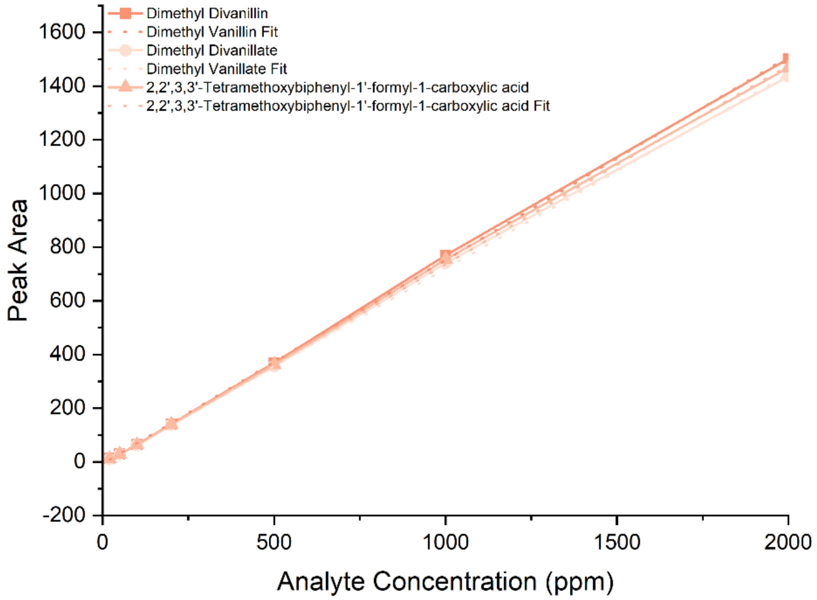

Supplementary Fig. 2. Calibration curves of dimethyl divanillin and dimethyl divanillate generated from analyte standards and the calculated curve of 2,2',3,3'-Tetramethoxybiphenyl-1'-formyl-1-carboxylic acid generated from the average peak areas of dimethyl divanillin and dimethyl divanillate.

Supplementary Table 1. Peak areas used averaged the calculated calibration curve of 2,2',3,3'-tetramethoxybiphenyl-1'-formyl-1-carboxylic acid.

Concentration (ppm)	Dimethyl divanillin (peak area)	Dimethyl divanillate (peak area)	2,2',3,3'-Tetramethoxybiphenyl-1'-formyl-1-carboxylic acid (calculated peak area)
2,000	1502.214	1435.843	1469.028
1,000	769.1883	740.7036	754.9459
500	369.006	355.7329	362.3695
200	140.2417	137.8494	139.0455
100	63.99691	62.2043	63.10061
50	28.85392	28.28121	28.56757
20	12.16792	10.32427	11.2461

Supplementary Table 2. Linear fit analysis of the calibration curves of dimethyl divanillin, dimethyl divanillate, and 2,2',3,3'-Tetramethoxybiphenyl-1'-formyl-1-carboxylic acid.

Dimethyl divanillin		Dimethyl divanillate		2,2',3,3'-Tetramethoxybiphenyl-1'-formyl-1-carboxylic acid	
Equation	y = a + b*x	Equation	y = a + b*x	Equation	y = a + b*x
Weight	No Weighting	Weight	No Weighting	Weight	No Weighting
Intercept	-7.01552 ± 4.62999	Intercept	-5.02723 ± 5.16852	Intercept	-6.02137 ± 4.87086
Slope	0.75834 ± 0.00532	Slope	0.7251 ± 0.00594	Slope	0.74172 ± 0.0056
Residual sum of squares	447.5708	Residual Sum of Squares	557.7419	Residual Sum of Squares	495.3507
Pearson's r	0.99988	Pearson's r	0.99983	Pearson's r	0.99986
R^2 (COD)	0.99975	R-Square (COD)	0.99966	R^2 (COD)	0.99972
Adj. R^2	0.9997	Adj. R^2	0.9996	Adj. R^2	0.99966

Gel permeation chromatography analysis

15-20 mg of lignin substrate was acetylated using 0.5 mL pyridine (Sigma-Aldrich anhydrous 99.8%) and 0.5 mL of acetic anhydride (Sigma-Aldrich reagent plus ≥ 99%) sealed and heated to 40°C for 24 hours while stirring. Subsequently, 1 mL aliquots of methanol were then added to each sample and dried under N_2. This was repeated five times. Samples are then dried under vacuum at 40 °C overnight. Samples are then diluted in THF and stirred for 30 minutes. The THF solution is filtered through a 0.2 μm syringe filter into an HPLC vial. 20 μL of sample was injected on an HPLC fitted with three PLgel 7.5 x 300 mm columns in series: 10 μm x 50 Å, 10 μm x 103 Å, 10 μm x 104 Å (Agilent Technologies, Stockport, UK) at ambient temperature with an isocratic 1 mL min-1 100% tetrahydrofuran (Sigma-Aldrich inhibitor-free ≥ 99.9%) for 45 minutes. Analytes were monitored at 210 nm, 260 nm, and 270 nm on the DAD.

^{31}P NMR of lignin substrates

Analysis of hydroxyl groups including aliphatic, phenolic, and carboxylic acids was performed on the lignin substrates using previously reported methods.[1,2] An internal standard solution of $CDCl_3$, triphenylphosphine oxide, and $Cr(acac)_3$ was prepared. To a 4 mL vial, was added 20-30 mg of the desired lignin substrate. Approximately 0.6 mL of the ISTD solution was then added to the sample, followed by 0.9 mL of anhydrous pyridine. Then, approximately 0.2 mL of 2-Chloro-4,4,5,5-tetramethyl-1,3,2-dioxaphospholane (TMDP) was added to each sample. The samples were capped, and shaken briefly to fully mix, and inspected to verify no precipitate had formed, and finally transferred into NMR tubes. ^{31}P NMR spectra were acquired on a Bruker Avance III HD Nanobay 400 MHz instrument equipped with a nitrogen-cooled Prodigy cryoprobe using an inverse gated decoupling pulse sequence, 25 second pulse delay, and 128 scans at 25 °C with a LB of 5.0 Hz. Processing (including phasing, baseline correction, and axis calibration) and quantification were performed using Bruker TopSpin 3.6 software. Supplementary Fig.s were generated using MestreNova. Samples were analyzed in duplicates.

Metabolite analysis by UHPLC

Bioconversion metabolites were analyzed using the previously methods.[13]

Derivatization of autoxidation products for GC-FID quantification

An aliquot of the autoxidation reaction mixture was taken, and the acetic acid was evaporated under a stream of N_2 at room temperature. Dimethylformamide, excess potassium carbonate (10 mass equiv), and methyl iodide (10 mass equiv), were then added to the residue, and the mixture was stirred at room temperature for 3 h. To the slurry was added an internal standard was added (naphthalene), and the mixture was filtered with a 0.2 μm porosity filter frit into an analysis vial before being analyzed by GC-FID. Validation of the derivatization on acid and aldehyde substrates is shown in **Supplementary Fig. 3**.

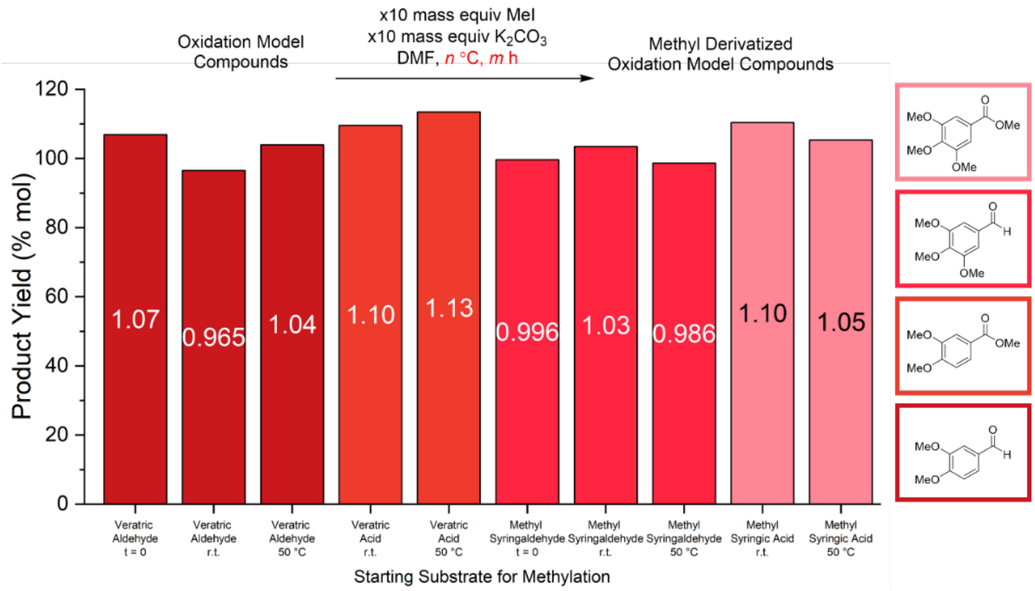

Supplementary Fig. 3. Verification of methylation protocol showing quantitative recovery of aldehyde products and carboxylic acids as their methyl ester derivative.

Synthesis, Oligomer Substrate Preparation, and Autoxidation Catalysis

Model Compound and Analyte Synthesis

1-(3′,4′,5′-trimethoxyphenyl)propane, 2. To a glass vial charged with 4-propylsyringol (1.0 g, 5.1 mmol), THF (15 mL), and a stir-bar, was added potassium carbonate (2.1 g, 15 mmol) and methyl iodide (4.3 g, 31 mmol). The mixture was stirred for 72 h at 50 °C until completion as indicated by thin-layer chromatography. After completion, water was added (20 mL) and then EtOAc (3 x 10 mL) for extraction. The combined extracts were dried over sodium sulphate and the volatiles were removed under rotary evaporation yielding an orange oil. Purification by flash column chromatography [silica, hexane/EtOAc (4:1)] afforded the title compound as a colorless oil (0.467 g, 46%). ^{1}H NMR (300 MHz, $CDCl_3$): δ 6.40 (s, 2H), 3.85 (s, 6H), 3.82 (s, 3H), 2.53 (t, J = 7.6 Hz, 2H), 1.63 (m, J = 7.6 Hz, 2H), 0.95 (t, J = 7.6 Hz, 3H) ppm. ^{13}C NMR (75 MHz, $CDCl_3$): δ 153.12, 138.63, 136.07, 105.40, 60.96, 56.14, 38.62, 24.77, 14.00 ppm. GC-EIMS (m/z): Calcd. 210.1 ([M]+); Found. 210.1. Analytical data matched that previously reported.[14]

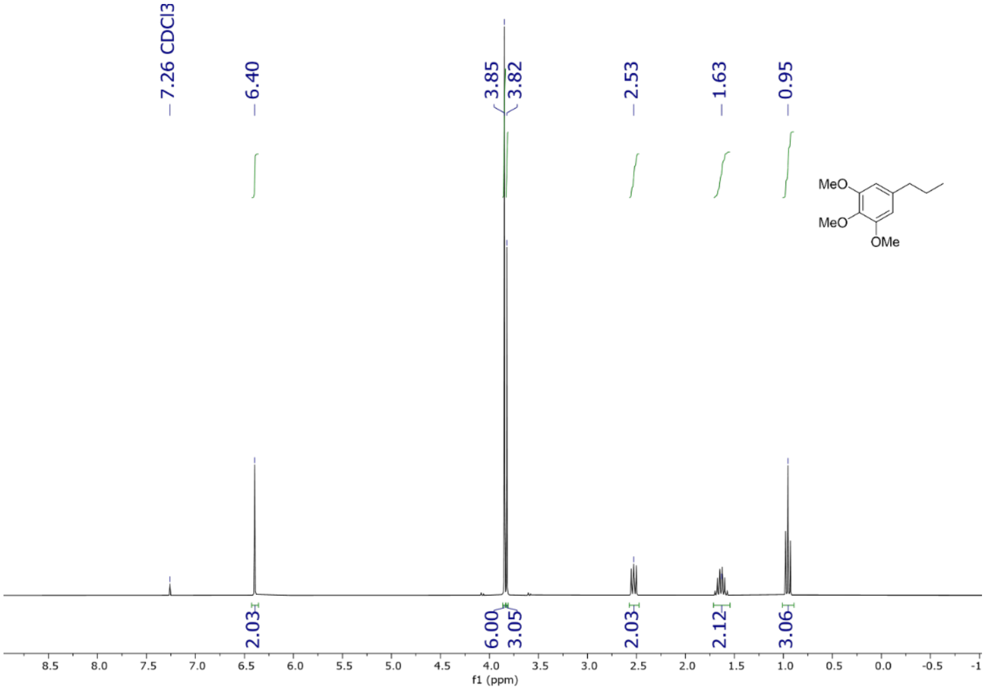

Supplementary Fig. 4. ^{1}H NMR spectrum of 1-(3′,4′,5′-trimethoxyphenyl)propane, **2**, in $CDCl_3$.

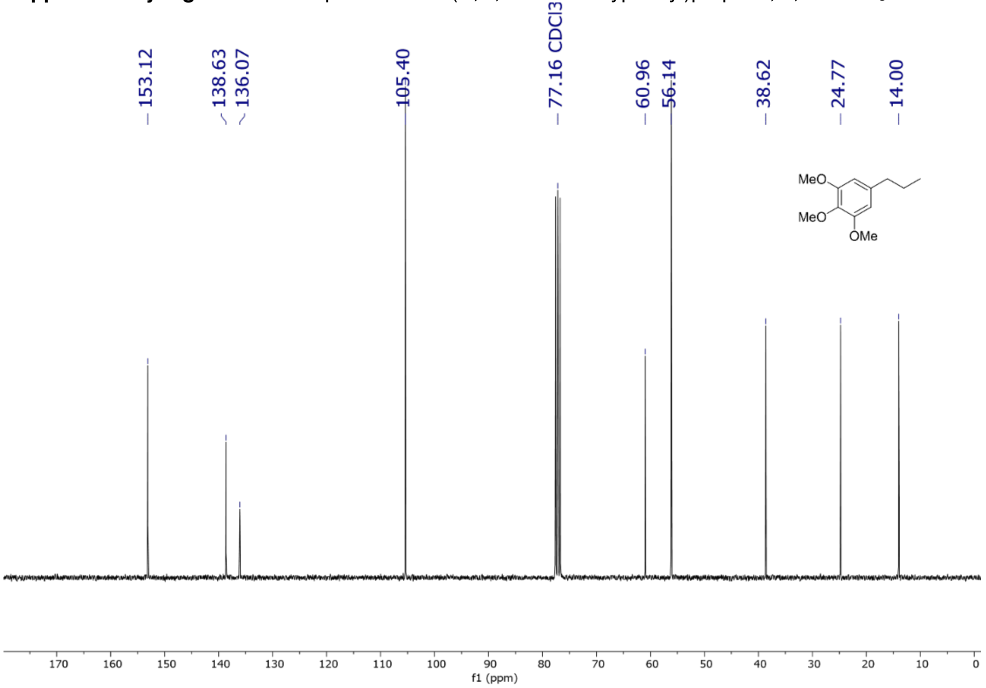

Supplementary Fig. 5. ^{13}C NMR spectrum of 1-(3′,4′,5′-trimethoxyphenyl)propane, **2**, in $CDCl_3$.

1,2-Bis(3,4-dimethoxyphenyl)ethane, 3. 1,2-bis(3,4-dimethylphenyl)ethylene was synthesized from 4-methoxy-isoeugenol through self cross-metathesis.[15] The 1,2-bis(3,4-dimethylphenyl)ethylene (504 mg, 1.68 mmol) obtained was hydrogenated in acetone/MeOH (3:1, v/v) (20 mL) with 10% Pd-C (100 mg) under H_2 atmosphere at ambient temperature for 2 hours to yield 1,2-bis(3,4-dimethylphenyl)ethane (495 mg, 97.6%). ^{1}H NMR (400 MHz, acetone-d_6): δ 6.83 (d, J = 8.2 Hz, 2H), 6.80 (s, 2H), 6.72 (d, J = 7.6 Hz, 2H), 3.76 (s, 12H), 2.83 (s, 4H) ppm. ^{13}C NMR (100 MHz, acetone-d_6): δ 150.38, 148.82, 135.61, 121.36, 113.79, 113.12, 56.31, 56.16, 38.44 ppm.

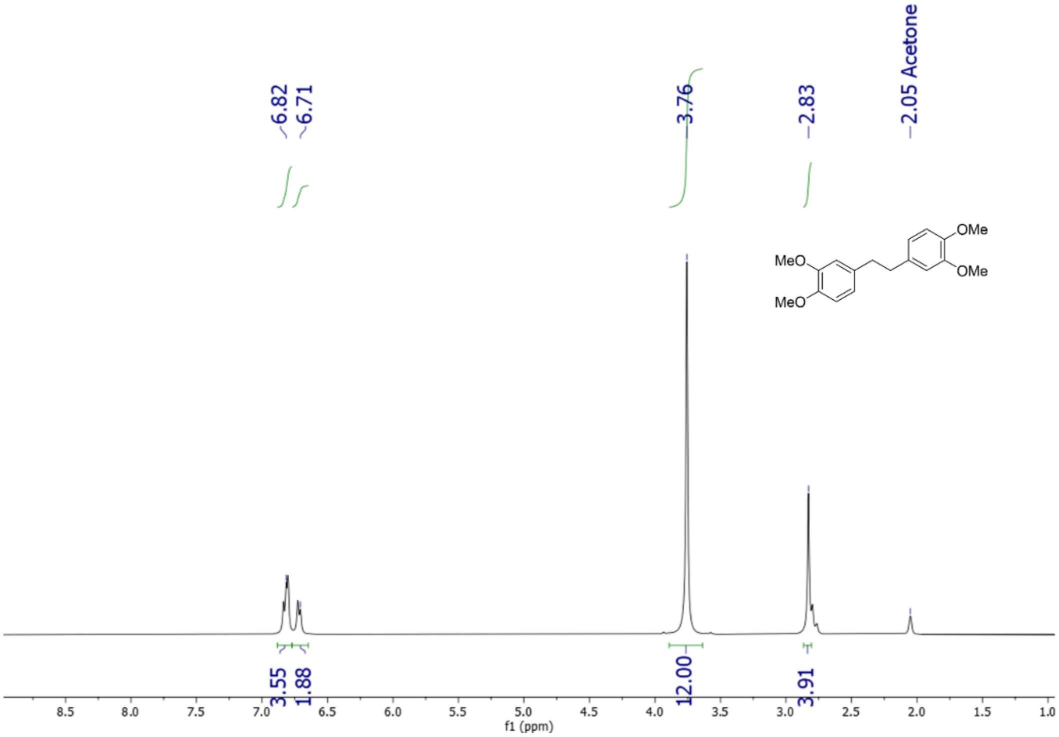

Supplementary Fig. 6. ^{1}H NMR spectrum of 1,2-bis(3,4-dimethoxyphenyl)ethane, **3**, in acetone-d_6.

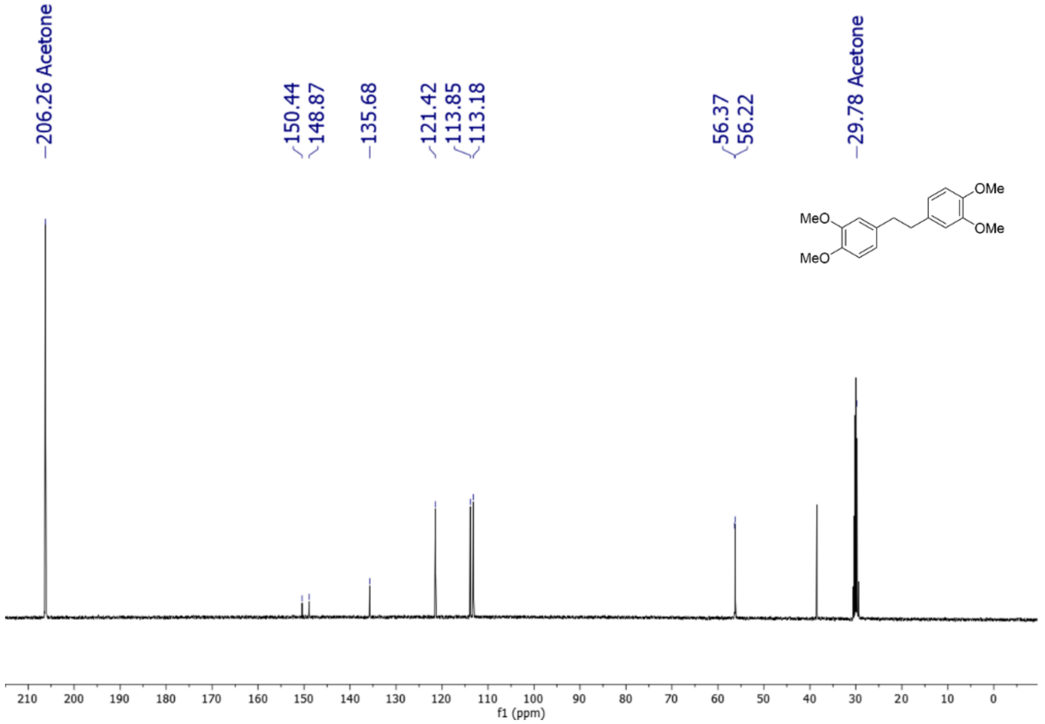

Supplementary Fig. 7. ^{13}C NMR spectrum of 1,2-bis(3,4-dimethoxyphenyl)ethane, **3**, in acetone-d_6.

Eudesmin, 4. To a glass vial charged with pinoresinol (169 mg, 0.472 mmol), acetonitrile (2 mL), and a stir-bar was added potassium carbonate (326 mg, 2.36 mmol) and methyl iodide (535 mg, 3.77 mmol). The mixture was stirred for 16 h at 50 °C. Water (10 mL) was added to the mixture, and it was extracted with EtOAc (3 x 5 mL). The combined extracts were dried over sodium sulphate and the volatiles were removed under rotary evaporation. Purification by flash column chromatography [silica, hexane/EtOAc (1:1)] afforded the title compound as a colorless solid (133 mg, 73%). ^{1}H NMR (300 MHz, $CDCl_3$): δ 6.91-6.85 (m, 6H), 4.77 (d, J = 4.2 Hz, 2H), 4.27 (dd, J = 6.9, 9.1, 2H), 3.90 (s, 6H), 3.88 (s, 3H), 3.12 (m, 2H) ppm. ^{13}C NMR (75 MHz, $CDCl_3$): δ 149.34, 148.77, 133.68, 118.38, 111.17, 109.35, 85.92, 71.86, 56.09, 56.06, 54.31 ppm. Analytical data matched that previously reported.[16]

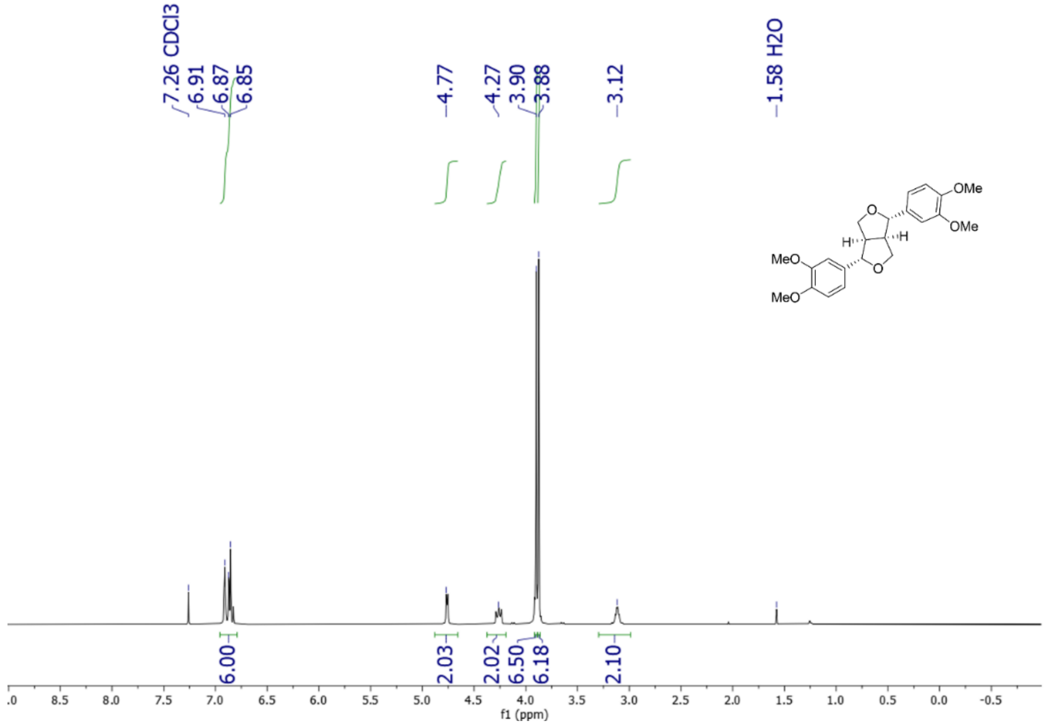

Supplementary Fig. 8. ^{1}H NMR spectrum of eudesmin, **4**, in $CDCl_3$.

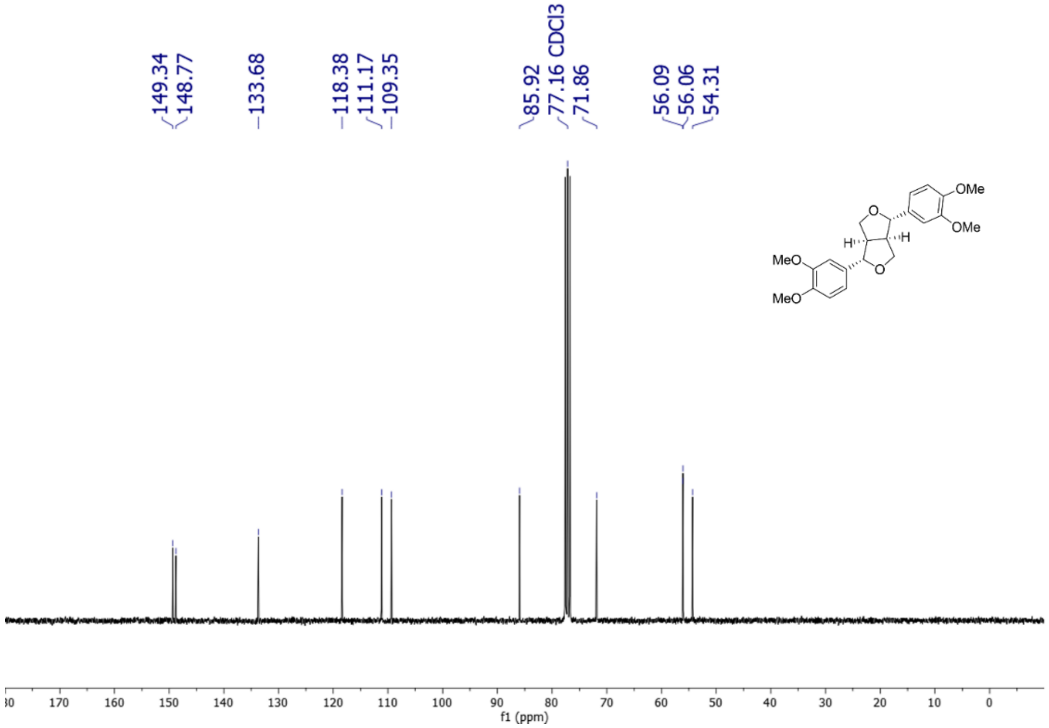

Supplementary Fig. 9. ^{13}C NMR spectrum of eudesmin, **4**, in $CDCl_3$.

1-(3,4-dimethoxyphenyl)-2-(2,3-dimethoxy-5-propyl-phenyl)-propane, 5. 1-(4-hydroxy-3-methoxy-phenyl)-2-(2-hydroxy-3-methoxy-5-propyl-phenyl)-propane was prepared according to Yue's paper.[17] To a solution of 1-(4-hydroxy-3-methoxy-phenyl)-2-(2-hydroxy-3-methoxy-5-propyl-phenyl)-propane (0.68 g, 2.05 mmol) in trimethylphosphate (4.81 mL, 41.1 mmol), potassium carbonate (0.57 g, 4.11 mmol) was added. The mixture was stirred for 0.5 h at 120 °C until completion as indicated by thin-layer chromatography. After completion, the mixture was filtered to remove excess amount of potassium carbonate. The filtrate was diluted with deionized H_2O (60 mL) and extracted with EtOAc (3 x 50 mL). The combined extracts were washed with brine, dried over sodium sulphate and the solvent was removed under rotary evaporation. Purification by flash column chromatography [silica, hexane/EtOAc (1:2)] afforded compound **5** as a white solid (121mg, 16.5%). ^{1}H NMR (400 MHz, $CDCl_3$): δ 6.73 (d, *J* = 8.2 Hz, 1H), 6.67 (dd, *J* = 8.8, 1.7 Hz, 2H), 6.57 (d, *J* = 1.7 Hz, 2H), 3.819 (s, 3H), 3.815 (s, 3H), 3.76 (s, 3H), 3.61 (s, 3H), 3.43 (m, *J* = 7.1 Hz, 1H), 2.87 (dd, *J* = 13.4, 6.9 Hz, 1H), 2.68 (dd, *J* = 13.4, 7.7 Hz, 1H), 2.53 (dd, *J* = 7.9, 7.4 Hz, 2H), 1.63 (sex, *J* = 7.4 Hz, 2H), 1.20 (d, *J* = 7.0 Hz, 3H), 0.95 (t, *J* = 7.4 Hz, 3H) ppm. ^{13}C NMR (75 MHz, $CDCl_3$): δ 152.31, 148.44, 147.10, 144.51, 140.01, 138.34, 133.92, 121.07, 118.69, 112.48, 110.90, 110.02, 60.67, 55.85, 55.65, 55.61, 43.84, 38.26, 34.37, 24.84, 20.98, 13.95 ppm. GC-EIMS (m/z): Calcd. 358.2 ([M]+); Found. 358.2.

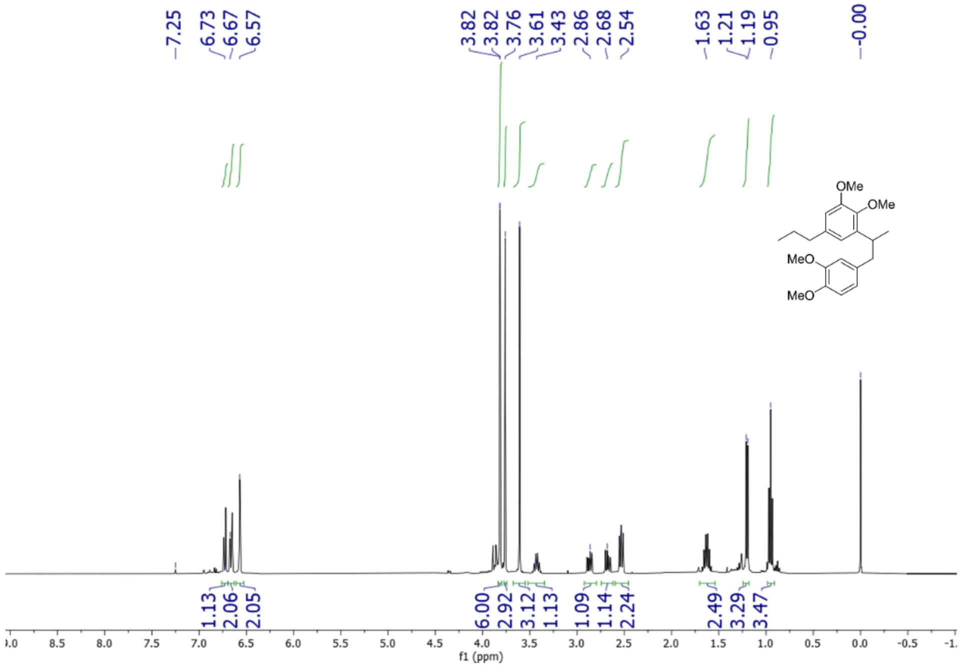

Supplementary Fig. 10. ^{1}H NMR spectrum of 1-(3,4-dimethoxy-phenyl)-2-(2,3-dimethoxy-5-propyl-phenyl)-propane, **5**, in $CDCl_3$.

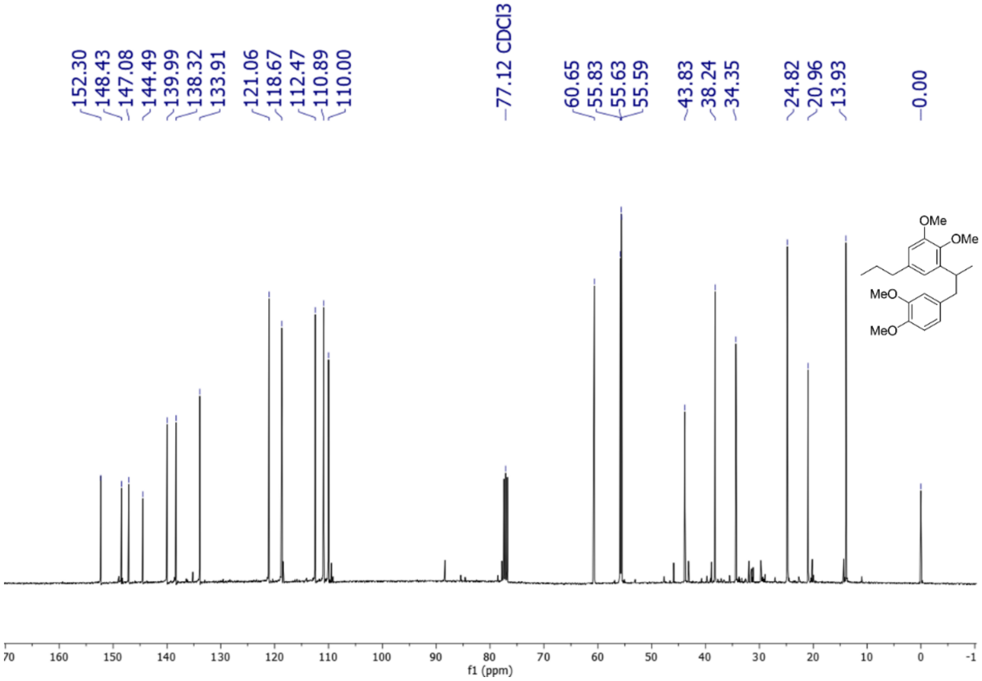

Supplementary Fig. 11. ^{13}C NMR spectrum of 1-(3,4-dimethoxy-phenyl)-2-(2,3-dimethoxy-5-propyl-phenyl)-propane, **5**, in $CDCl_3$.

2-Acetoxypropioveratrone. In a manner similar to that described,[18] acetyl chloride (493 mg, 6.27 mmol), was slowly added to a solution of 2-hydroxypropioveratrone[7] (440 mg, 2.01 mmol) and pyridine (497 mg, 6.27 mg) at 0 °C in CH_2Cl_2 (5 mL) under N_2. The mixture was left to warm to room temperature and stir for 1.5 h. The volatiles were removed under reduced pressure, and to the oil was added EtOAc (5 mL). The mixture was washed with H_2O (20 mL) and saturated aq. $NaHCO_3$ (20 mL) and subsequently dried over sodium sulphate. Removal of the volatiles under reduced pressure gave an oil that upon purification by flash column chromatography [silica, hexane/EtOAc (1:1)] gave the title compound as a colorless solid (308 mg, 58%). ^{1}H NMR (300 MHz, $CDCl_3$): δ 7.58, (dd, *J* = 2.0, 8.4 Hz, 1H), 7.55 (d, *J* = 2.0 Hz, 1H), 6.89 (d, *J* = 8.4 Hz, 1H), 5.94 (q, *J* = 7.0 Hz, 1H), 3.94 (s, 3H), 3.92 (s, 3H), 2.13 (s, 3H), 1.51 (d, *J* = 7.0 Hz, 3H) ppm. ^{13}C NMR (75 MHz, $CDCl_3$): δ 195.40, 170.56, 153.87, 148.88, 127.46, 122.88, 112.65, 110.24, 71.11, 56.23, 56.10, 21.61, 18.05 ppm. GC-EIMS (m/z): Calcd. 252.1 ([M]+); Found. 252.1.

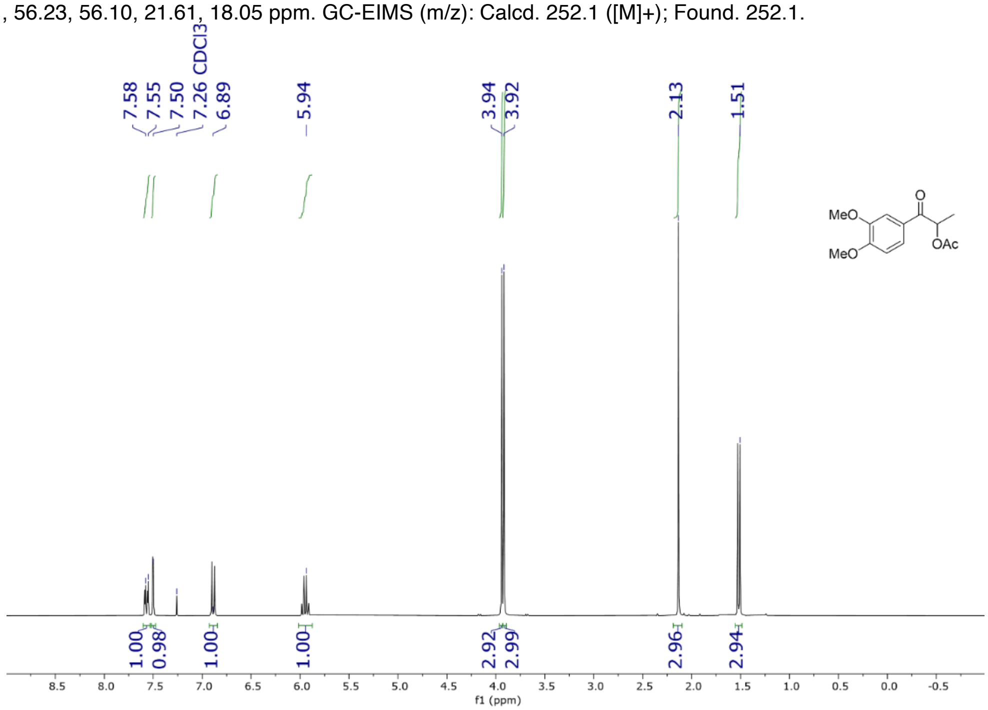

Supplementary Fig. 12. ^{1}H NMR spectrum of 2-Acetoxypropioveratrone in $CDCl_3$.

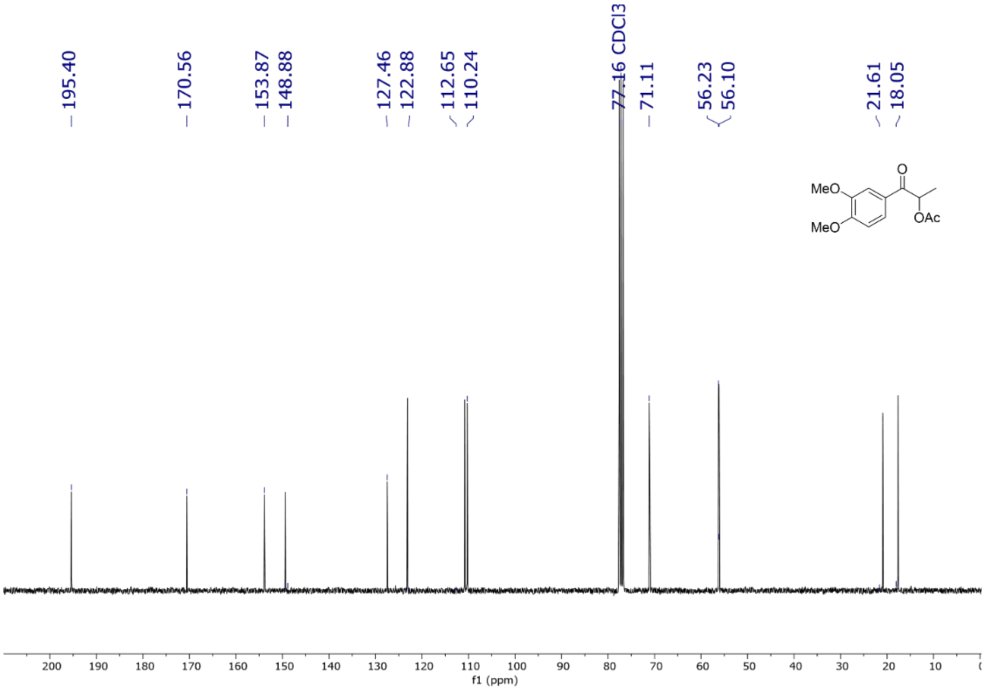

Supplementary Fig. 13. ^{13}C NMR spectrum of 2-Acetoxypropioveratrone in $CDCl_3$.

2-Hydroxy-1-(3′,4′,5′-trimethoxyphenyl)-propan-1-one. Following the previously reported method,[7] to a glass vial charged with 1-(3′,4′,5′-trimethoxyphenyl)propan-1-one (102 mg, 0.454 mmol), iodine (23 mg, 0.091 mmol), and a magnetic stir bar was added DMSO (1 mL), and the mixture was stirred at 60 °C. After 24 h, the mixture was diluted with EtOAc (5 mL), washed with 0.1 M $Na_2S_2O_3$ (5 mL), extracted with EtOAc (3 x 2 mL), and the volatiles were removed under vacuum. The crude product was purified by column chromatography [silica, hexane/EtOAc (9:1) to yield 2-hydroxy-1-(3,4,5-trimethoxyphenyl)-propan-1-one (34 mg, 31%). ^{1}H NMR (300 MHz, CD_2Cl_2): δ 7.17 (s, 2H), 5.13 (q, *J* = 7.0 Hz, 1H), 3.89 (s, 6H), 3.86 (s 3H), 3.68 (br, 1H), 1.42 (d, *J* = 7.0 Hz, 3H) ppm. ^{13}C NMR (75 MHz, CD_2Cl_2): δ 201.77, 153.75, 143.73, 128.90, 106.52, 69.44, 60.97, 22.81 ppm.

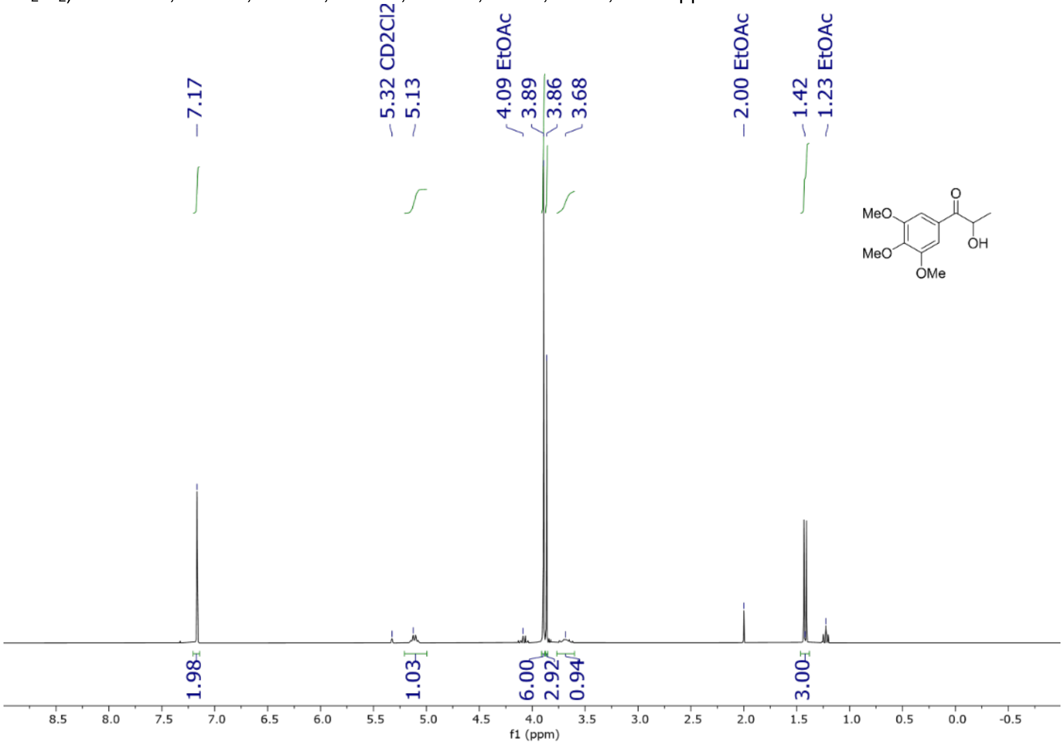

Supplementary Fig. 14. ^{1}H NMR spectrum of 2-hydroxy-1-(3,4,5-trimethoxyphenyl)-propan-1-one in CD_2Cl_2.

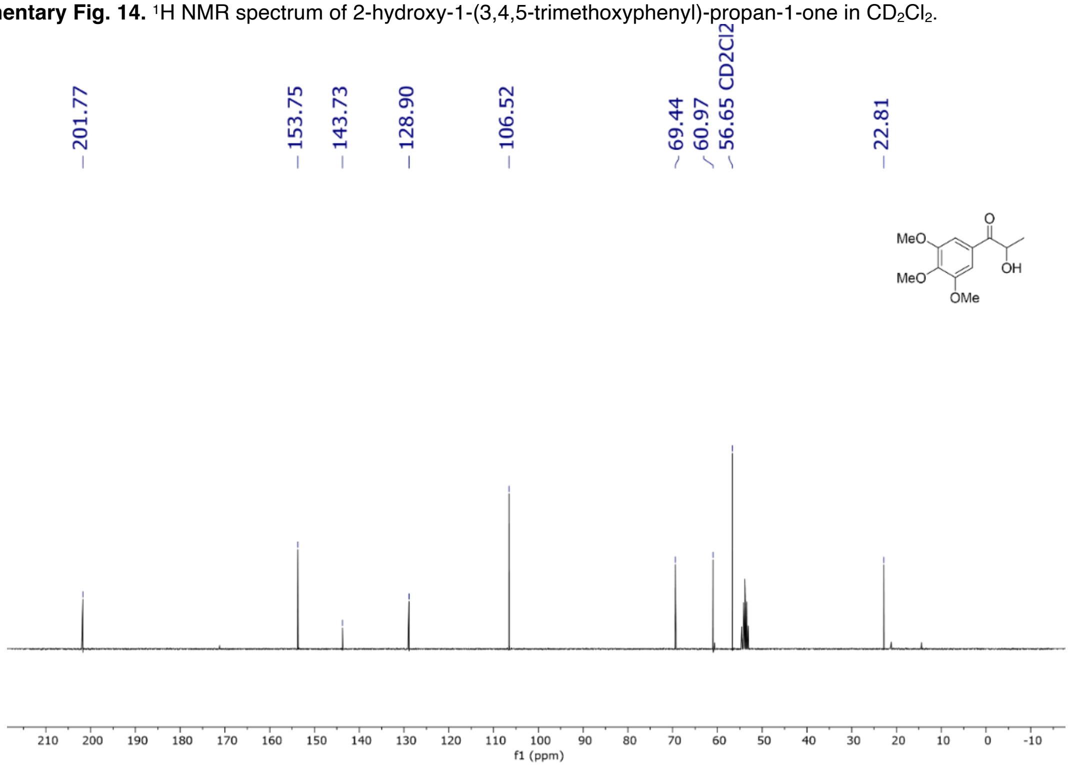

Supplementary Fig. 15. ^{13}C NMR spectrum of 2-hydroxy-1-(3,4,5-trimethoxyphenyl)-propan-1-one in CD_2Cl_2.

2-Acetoxy-1-(3′,4′,5′-trimethoxyphenyl)-propan-1-one. To a stirred solution of 2-hydroxy-1-(3,4,5-trimethoxyphenyl)-propan-1-one (34 mg, 0.14 mmol) in CH_2Cl_2 (1 mL) was added acetic anhydride (27 μL, 0.28 mmol) and pyridine (46 μL, 0.57 mmol). The mixture was stirred at room temperature for 36 h, concentrated, and purified by column chromatography [silica, hexane/EtOAc (1:1)] gave 2-Acetoxy-1-(3′,4′,5′-trimethoxyphenyl)-propan-1-one (30 mg, 75%). ^{1}H NMR (300 MHz, $CDCl_3$): δ 7.20 (s, 2H), 5.95 (q, J = 7.0 Hz, 1H), 3.92 (s, 3H), 3.91 (s, 6H), 2.15 (s, 3H), 1.53 (d, J = 7.0 Hz, 3H) ppm. ^{13}C NMR (75 MHz, $CDCl_3$): δ 195.78, 170.63, 153.36, 143.24, 129.59, 106.16, 71.16, 61.11, 56.48, 20.92, 17.45 ppm. GC-EIMS (m/z): Calcd. 282.1 ([M]+); Found. 282.1

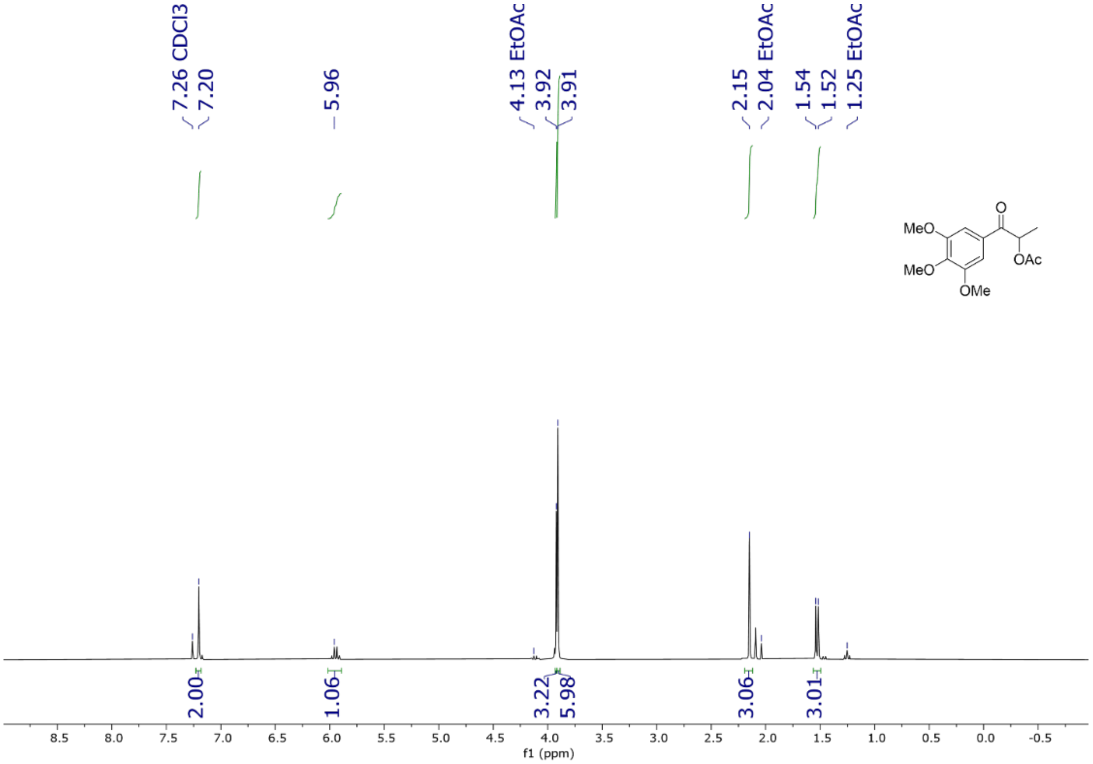

Supplementary Fig. 16. ^{1}H NMR spectrum of 2-acetoxy-1-(3,4,5-trimethoxyphenyl)-propan-1-one in $CDCl_3$.

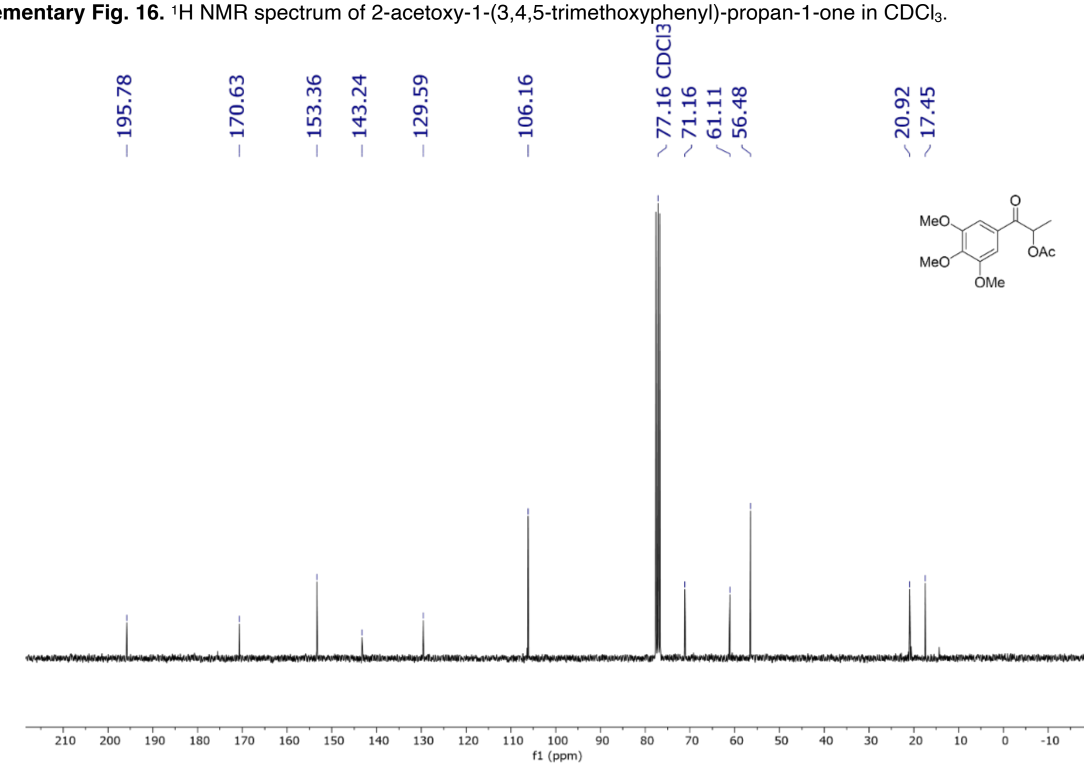

Supplementary Fig. 17. ^{13}C NMR spectrum of 2-acetoxy-1-(3,4,5-trimethoxyphenyl)-propan-1-one in $CDCl_3$.

Dimethyl 3,4,-dimethoxy-1,5-benzenedicarboxylate. A pressure-rated reaction tube was charged with 4-hydroxy-5-methoxyisophthalic acid (1.0 g, 4.7 mmol), DMF (50 mL), and a stir-bar, was added potassium carbonate (1.95 g, 14.1 mmol) and methyl iodide (4.0 g, 28.3 mmol). The mixture was stirred for 24 h at 80 °C. After cooling to room temperature, water (50 mL) was added to the mixture, which was extracted with EtOAc (3 x 20 mL). The combined organic extracts were dried over sodium sulphate. Removal of the volatiles under reduced pressure gave an oil that upon purification by flash column chromatography [silica, hexane/EtOAc (4:1)] gave the title compound as a colorless solid (1.034 g, 86%). ^{1}H NMR (300 MHz, $CDCl_3$) δ 8.01 (s, 1H), 7.68 (s, 1H), 3.94 (s, 3H), 3.92 (s, 3H), 3.91 (s, 3H), 3.90 (s, 3H) ppm. ^{13}C NMR (75 MHz, $CDCl_3$): δ 166.09, 166.02, 153.47, 151.94, 126.35, 125.62, 124.28, 116.18, 61.75, 56.33, 52.48, 52.42 ppm. GC-EIMS (m/z): Calcd. 254.1 ([M]+); Found. 254.1. Analytical data matches that previously reported.[19]

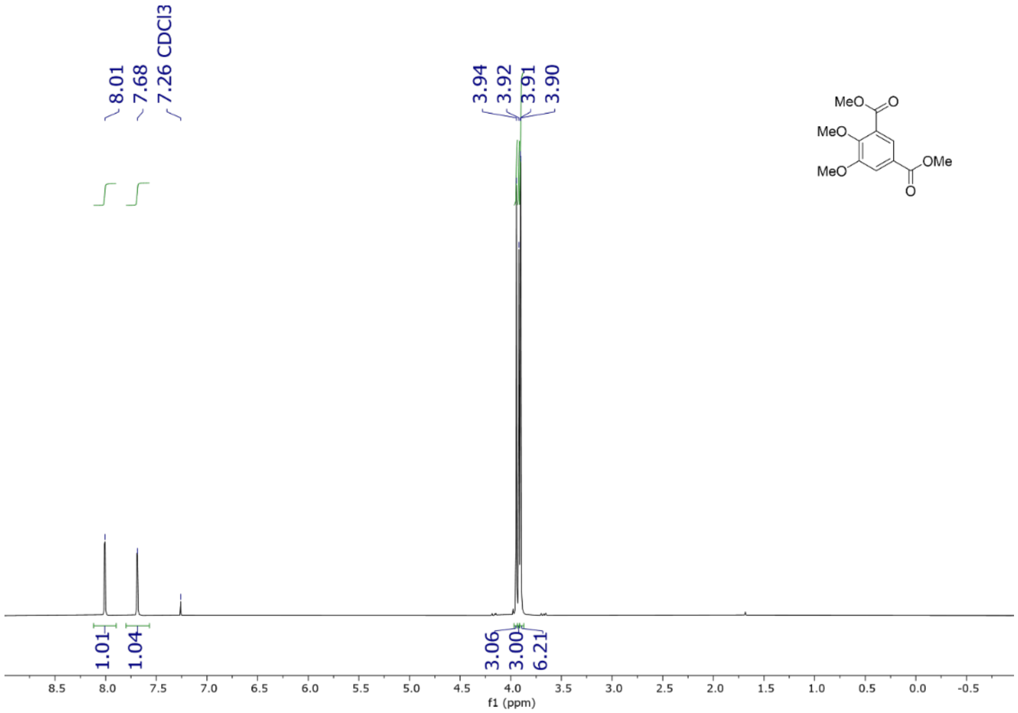

Supplementary Fig. 18. ^{1}H NMR spectrum of dimethyl 3,4,-dimethoxy-1,5-benzenedicarboxylate in $CDCl_3$.

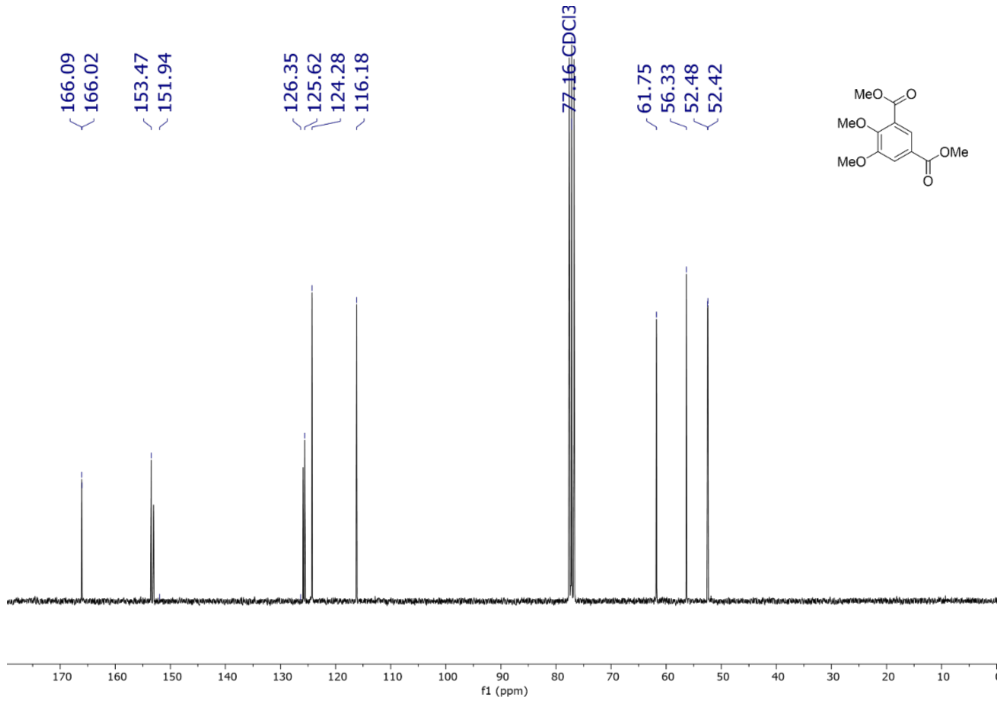

Supplementary Fig. 19. ^{13}C NMR spectrum of dimethyl 3,4,-dimethoxy-1,5-benzenedicarboxylate in $CDCl_3$.

3,4-Dimethoxy,1,5-benzenedicarboxylic acid. A glass vial was charged with dimethyl 3,4,-dimethoxy-1,5-benzenedicarboxylate (200 mg, 0.00787 mmol), a solution of potassium hydroxide (284 mg, 5.06 mmol) in methanol (2 mL), and a magnetic stir-bar. The mixture was stirred at room temperature for 48 h. To the resultant mixture was added deionized water (2 mL), and aqueous hydrochloric acid until pH = 2. The colorless solid was collected by filtration, washed with deionized water, and dried under reduced pressure to yield the title compound as a colorless solid (123 mg, 69%). ^{1}H NMR (300 MHz, DMSO-d_6): δ 13.10 (br s, 2H), 7.79 (d, J = 2.0 Hz, 1H), 7.65 (d, J = 2.0 Hz, 1H), 3.89 (s, 3H), 3.81 (s, 3H) ppm. ^{13}C NMR (300 MHz, DMSO-d_6): δ 166.63, 166.27, 152.96, 151.42, 126.96, 126.19, 122.86, 115.47, 60.99, 56.04 ppm.

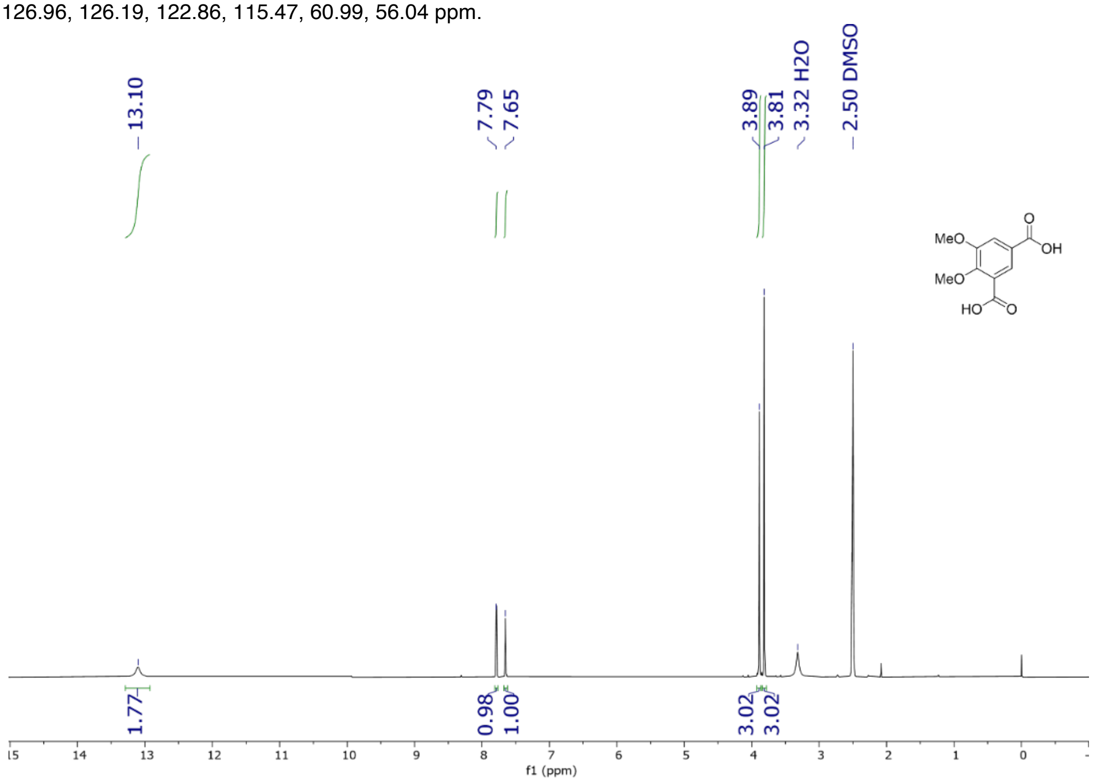

Supplementary Fig. 20. ^{1}H NMR spectrum of 3,4-dimethoxy,1,5-benzenedicarboxylic acid in DMSO-d_6.

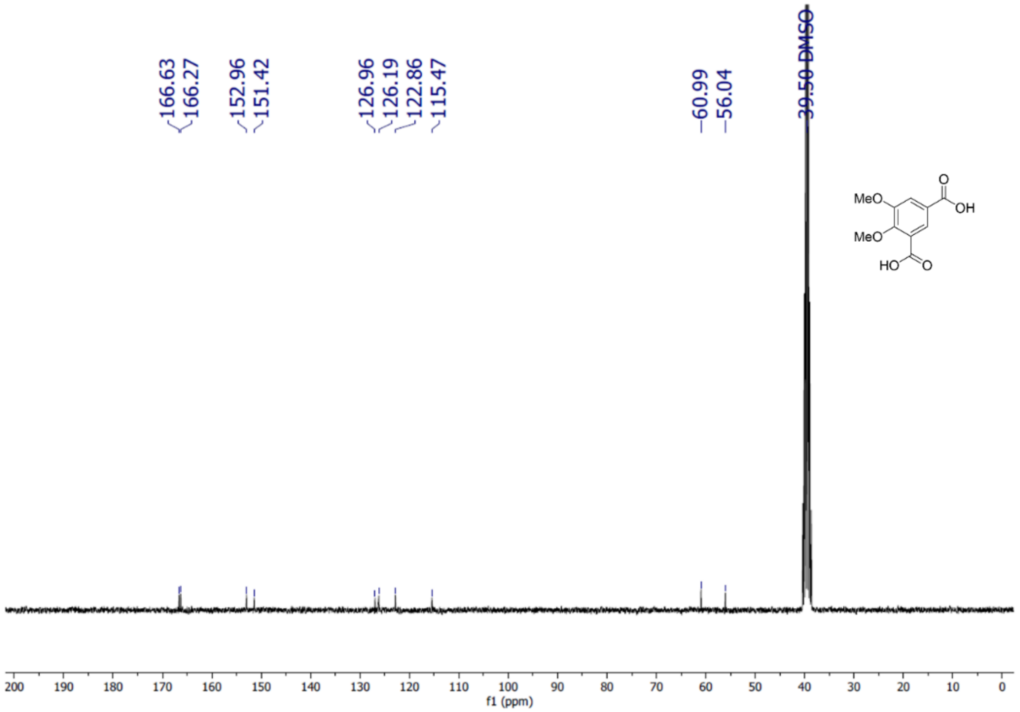

Supplementary Fig. 21. ^{13}C NMR spectrum of 3,4-dimethoxy,1,5-benzenedicarboxylic acid in DMSO-d_6.

Isoelemicin. 4-Propyenylsyringol (0.12 g, 6.18 mmol) was methylated in the same method as compound **5** in trimethylphosphate (1.45 mL, 12.4 mmol) with potassium carbonate (0.17 g, 1.24 mmol). The crude reaction mixture was purified by flash column chromatography [silica, hexane/EtOAc (1:2)] to get isoelemicin as a colorless solid (50.1 mg, 38.9%). Analytical data matched that previously reported.[20] ^{1}H NMR (400 MHz, $CDCl_3$): δ 6.55 (s, 2H), 6.33 (dd, *J* = 15.7, 1.6 Hz, 1H), 6.15 (dq, *J* = 15.6, 6.6 Hz, 1H), 3.86 (s, 6H), 3.83 (s, 3H), 1.87 (dd, *J* = 6.6, 1.6 Hz, 3H) ppm. ^{13}C NMR (75 MHz, $CDCl_3$): δ 153.26, 137.14, 133.77, 130.90, 125.30, 102.81, 56.01, 18.37 ppm. Quantitative ^{1}H NMR indicates 88% purity. Attempts for further purification were unsuccessful.

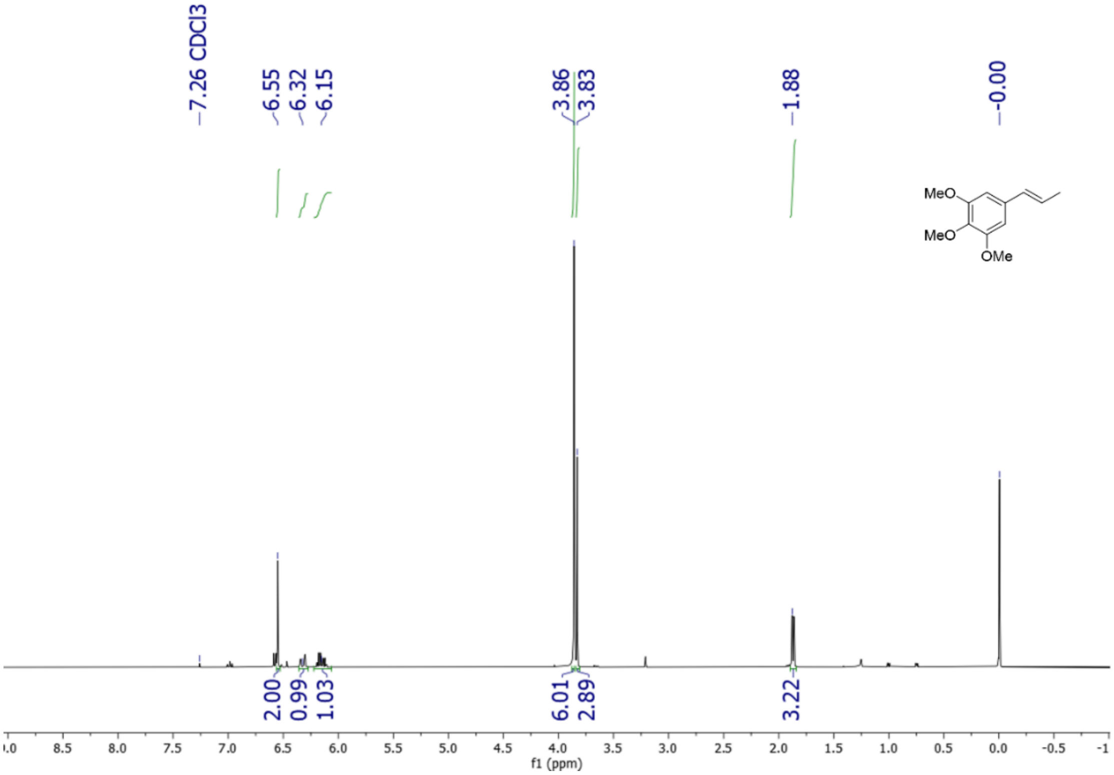

Supplementary Fig. 22. ^{1}H NMR spectrum of isoelemicin in $CDCl_3$.

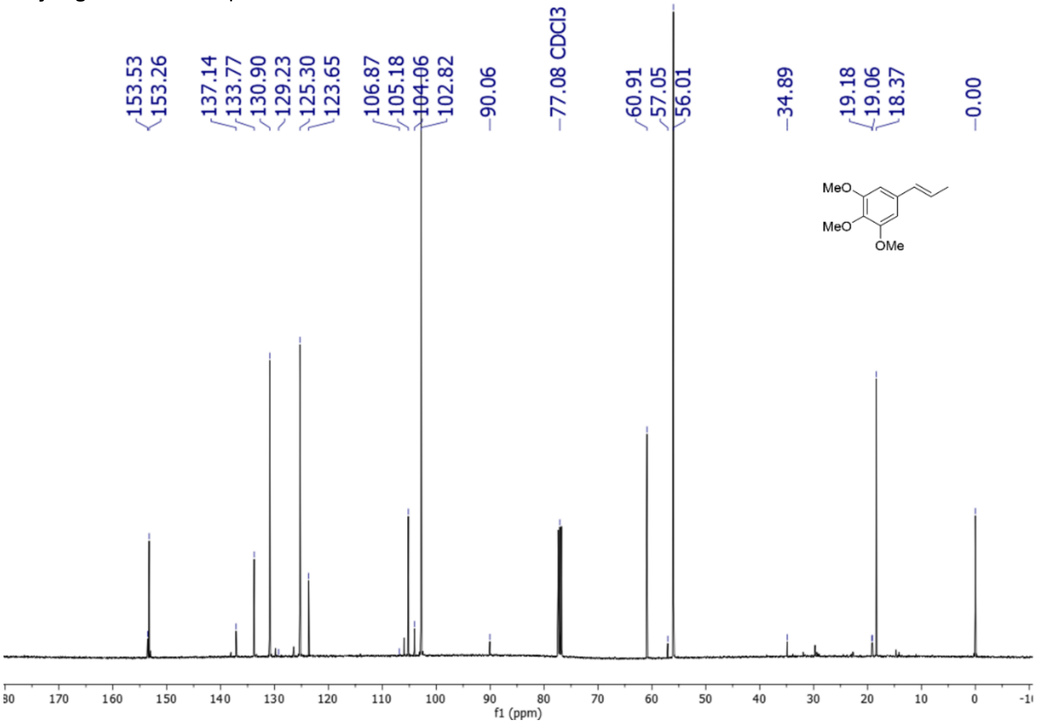

Supplementary Fig. 23. ^{13}C NMR spectrum of isoelemicin in $CDCl_3$.

1-(3′,4′-dimethoxyphenyl)-3-methoxypropane. Dihydroconiferyl alcohol (0.2g, 1.10 mmol) was methylated in the same method as compound **4** in trimethylphosphate (2.57 ml, 22.0 mmol) with potassium carbonate (0.30 g, 2.20 mmol). The crude reaction mixture was purified by flash column chromatography [silica, hexane/EtOAc (1:2)] to get 1-(3′,4′-dimethoxyphenyl)-3-methoxypropane (155 mg, 67.2%). ^{1}H NMR (400 MHz, $CDCl_3$): δ 6.78 (d, *J* = 8.6 Hz, 1H), 6.71 (m, 2H), 4.14 (t, *J* = 6.5 Hz, 1H), 3.85 (s, 3H), 3.83 (s, 3H), 3.77 (s, 3H), 2.65 (dd, *J* = 8.0, 7.4 Hz, 1H), 1.96 (m, 1H) ppm. ^{13}C NMR (75 MHz, $CDCl_3$): δ 148.85, 147.29, 133.50, 120.18, 111.64, 111.24, 67.22, 55.81, 55.71, 54.59, 31.407, 30.413 ppm.

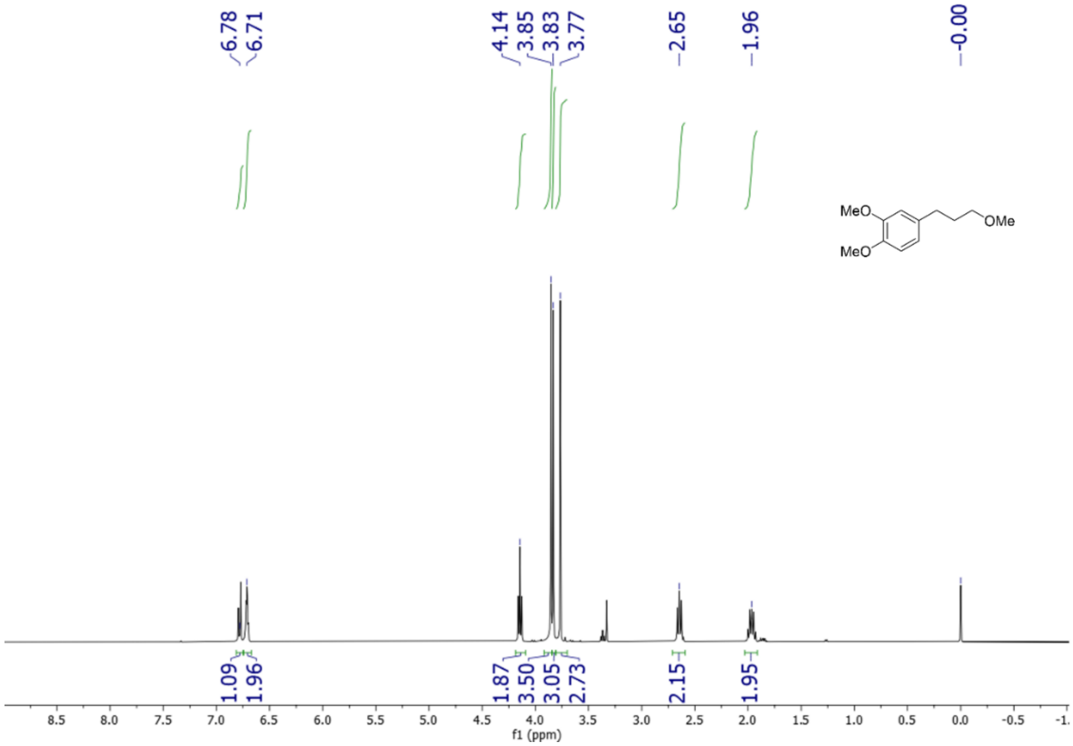

Supplementary Fig. 24. ^{1}H NMR spectrum of 1-(3′,4′-dimethoxyphenyl)-3-methoxypropane in $CDCl_3$.

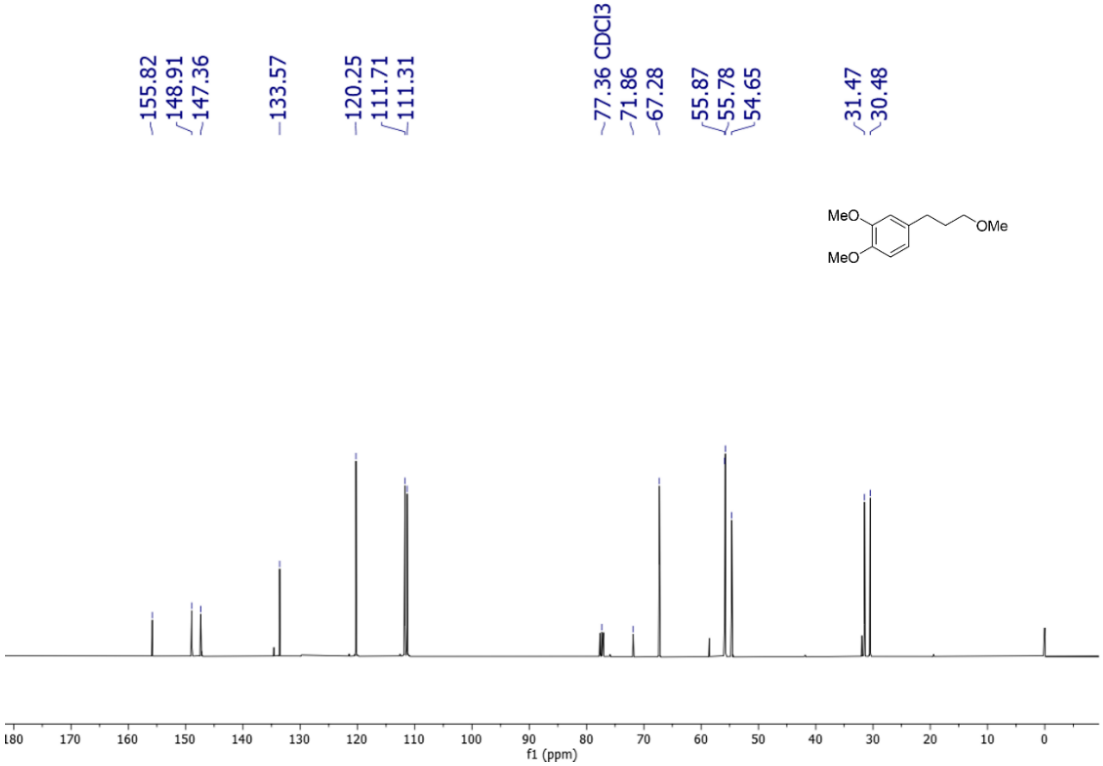

Supplementary Fig. 25. ^{13}C NMR spectrum of 1-(3′,4′-dimethoxyphenyl)-3-methoxypropane in $CDCl_3$.

1-(3′,4′,5′-trimethoxyphenyl)-3-methoxypropane. Dihydrosinapyl alcohol (0.2 g, 0.94 mmol) was methylated in the same method as compound **4** in trimethylphosphate (2.21 ml, 18.8 mmol) with potassium carbonate (0.26 g, 1.88 mmol). The crude reaction mixture was purified by flash column chromatography [silica, hexane/EtOAc (1:2)] to yield 1-(3′,4′,5′-trimethoxyphenyl)-3-methoxypropane (163 mg, 71.8%). ^{1}H NMR (400 MHz, $CDCl_3$): δ 6.42 (s, 2H), 4.17 (t, *J* = 6.4 Hz, 1H), 3.84 (s, 6H), 3.82 (s, 2H), 3.78 (s, 3H), 2.65 (dd, *J* = 8.0, 7.4 Hz, 1H), 1.99 (m, 1H) ppm. ^{13}C NMR (75 MHz, $CDCl_3$): δ 155.83, 153.21, 136.80, 136.24, 105.29, 67.28, 60.77, 56.01, 54.69, 32.33, 30.38 ppm.

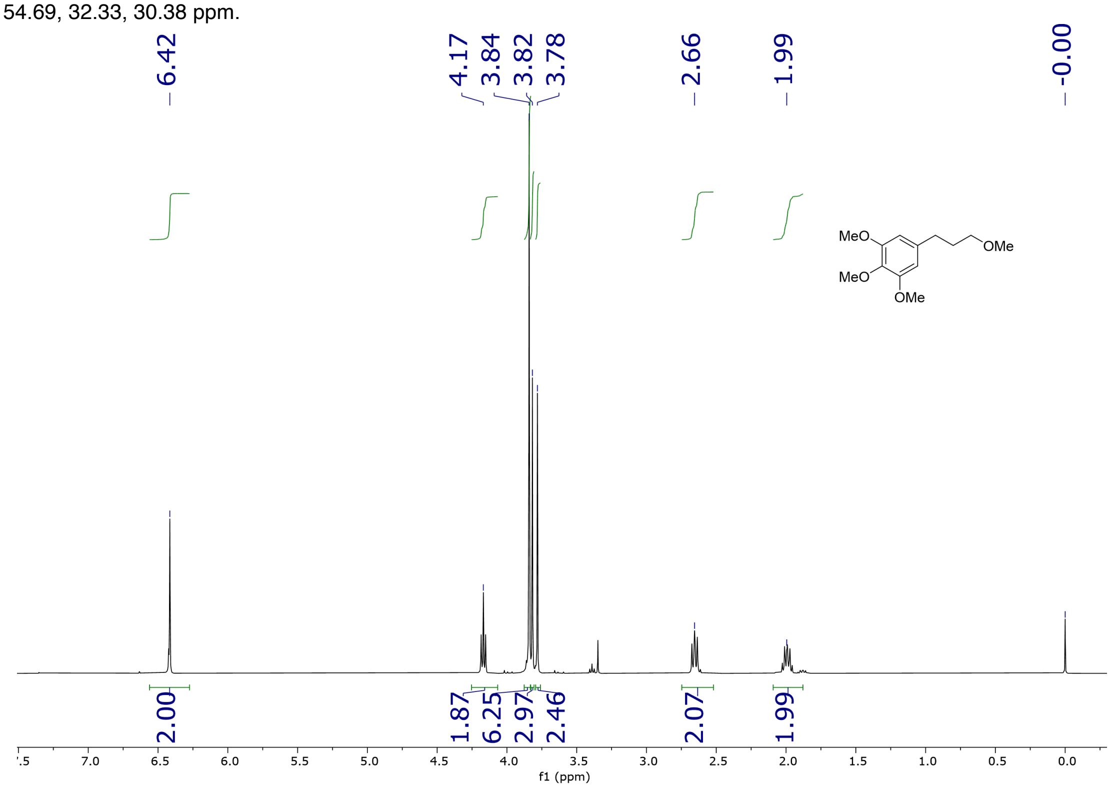

Supplementary Fig. 26. ^{1}H NMR spectrum of 1-(3′,4′,5′-trimethoxyphenyl)-3-methoxypropane in $CDCl_3$.

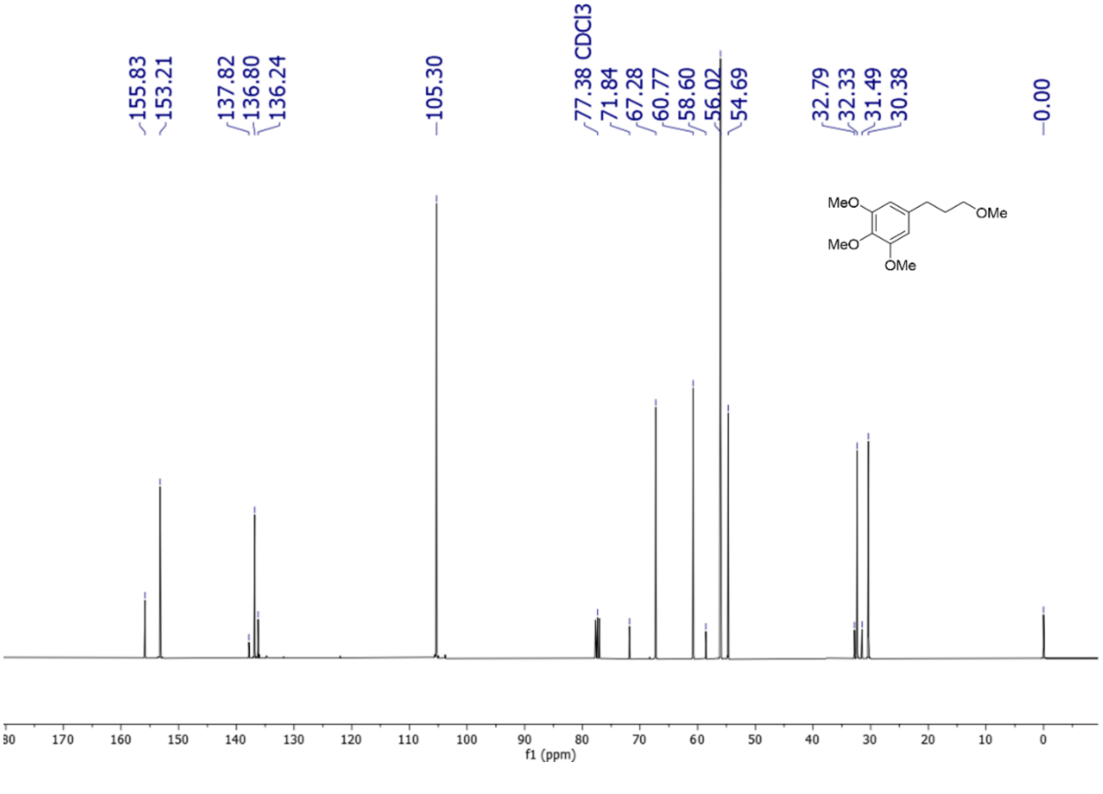

Supplementary Fig. 27. ^{13}C NMR spectrum of 1-(1′,2′,6′-trimethoxyphenyl)-3-methoxypropane in $CDCl_3$.

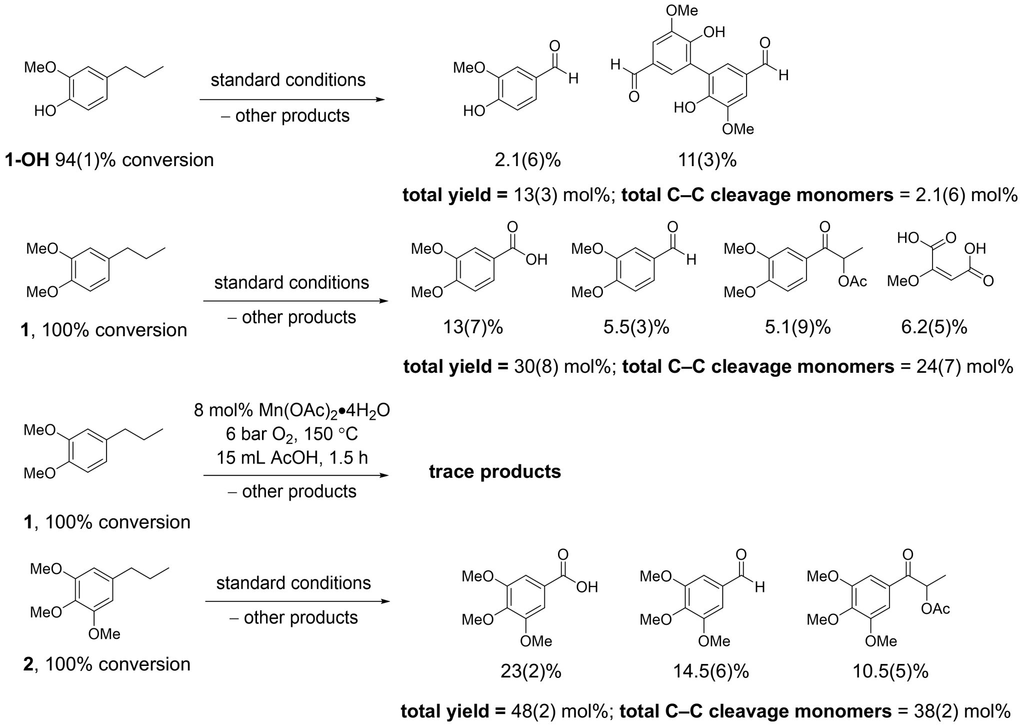

Supplementary Fig. 28. Autoxidation of monomer models **1-OH**, **1**, and **2**, with yields shown as value(standard deviation) in mol% aromatics. Standard conditions: substrate, 0.1 mmol; catalyst, 8 mol% $Mn(OAc)_2$•$4H_2O$, 6 mol% $Zr(acac)_4$; solvent, 15 mL acetic acid; O_2 loading, 6 bar; time, 1.5 h; temperature, 150 °C.

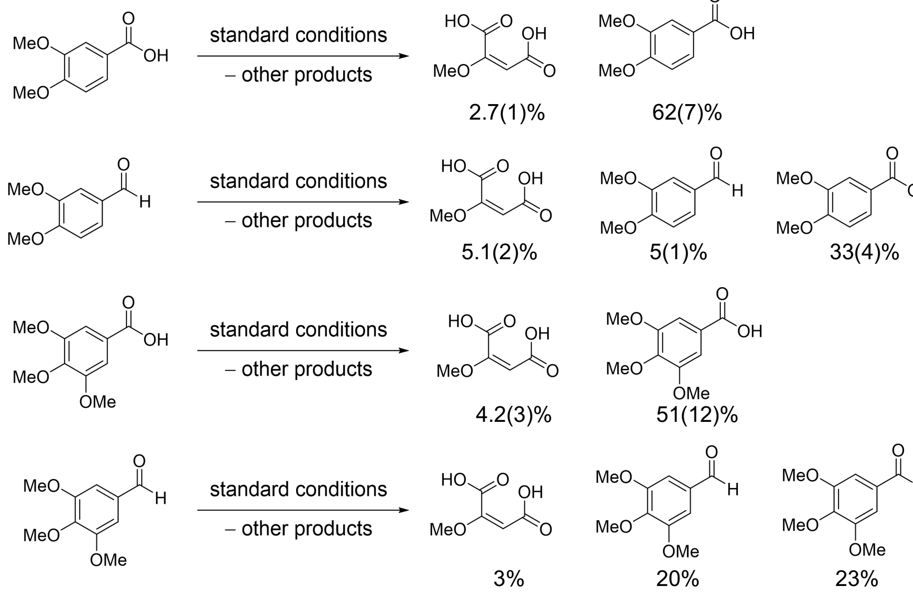

Supplementary Fig. 29. Product stability reactions under autoxidation conditions with yields shown as value(standard deviation) in mol% aromatics. Standard conditions: substrate, 0.1 mmol; catalyst, 8 mol% $Mn(OAc)_2$•$4H_2O$, 6 mol% $Zr(acac)_4$; solvent, 15 mL acetic acid; O_2 loading, 6 bar; time, 1.5 h; temperature, 150 °C.

Autoxidation of the Model Compounds

For a single reaction, a 75 mL Parr batch reactor fit with a glass liner insert was charged with 0.1 mmol of substrate, acetic acid (15 mL), a stir bar, and 8 mol% $Mn(OAc)_2$•$4H_2O$ and 6 mol% $Zr(acac)_4$ catalyst. The mixture was pressurized three times with pure nitrogen and subsequently charged with air and nitrogen to achieve the desired oxygen partial pressure. The vessel was heated to temperature, at which, it was maintained for the desired timeframe before it was cooled rapidly in an ice bath. The solutions were stored in the

freezer until needed for analysis. Unless noted, the model compound reactions were run in triplicate. 4-Propylveratrole, **1**, and 1,2-bis(3,4-dimethoxyphenyl)ethane, **3,** veratraldehyde, veratric acid, and syringic acid were run in duplicate.

Oligomer Substrate Preparation

Reductive catalytic fractionation. The reported procedure[21] was performed with poplar and pine biomass: To a 11.4 L Parr batch reactor vessel was added 5% Ru/C (15 g) and deionized water (15 g). The vessel was connected to a supply of dinitrogen and the vessel was purged for 5 min. Under a flow of dinitrogen, methanol was added (3 L) slowly to not agitate the catalyst. The biomass (300 g) was added slowly, and the reaction vessel was stirred manually in order to suspend the biomass in the methanol. The flux of nitrogen was removed, and the reactor head was immediately installed. The vessel was pressure tested with dinitrogen (117 bar), where the maximum acceptable loss was 7 mbar/min. Subsequently, the reactor was flushed twice with 27.6 bar of dinitrogen to sparge the vessel of any residual air. Once all the dinitrogen was drained, the vessel was pressured with dihydrogen (30 bar). The mixture was then heated (225 °C) with the mag drive set to 80% of max stirring, and cooling water was run through the mag drive. After 6 h, the vessel was rapidly cooled with water through a cooling coil. The vessel was depressurized, and the product was pumped out of the mixture using a peristaltic pump. The mixture was filtered, and the methanol was removed by rotary evaporation. The residual oil was extracted with equivolume ethyl acetate and deionized water. The water fraction was extracted two more times with ethyl acetate and the ethyl acetate layers were combined, washed with water, dried with sodium sulfate, and ethyl acetate was evaporated by rotary evaporation.

Methylation of pine RCF oil. A 250 mL round-bottom flask was charged with pine RCF oil (7.869 g), potassium carbonate (30.0 g, 0.211 mol), acetonitrile (125 mL), and a stir-bar. Methyl iodide (61.1 g, 0.441 mol) was added, and the solution was stirred at room temperature. After 24 h, to the resultant brown slurry was added water (200 mL), and the mixture was extracted with ethyl acetate (3 x 150 mL). The organic layers were combined and washed with water (2 x 100 mL) and brine (1 x 100 mL). The organic layer was subsequently dried with sodium sulphate, filtered, and the volatiles were removed by rotary evaporation leaving a brown viscous oil (8.381 g).

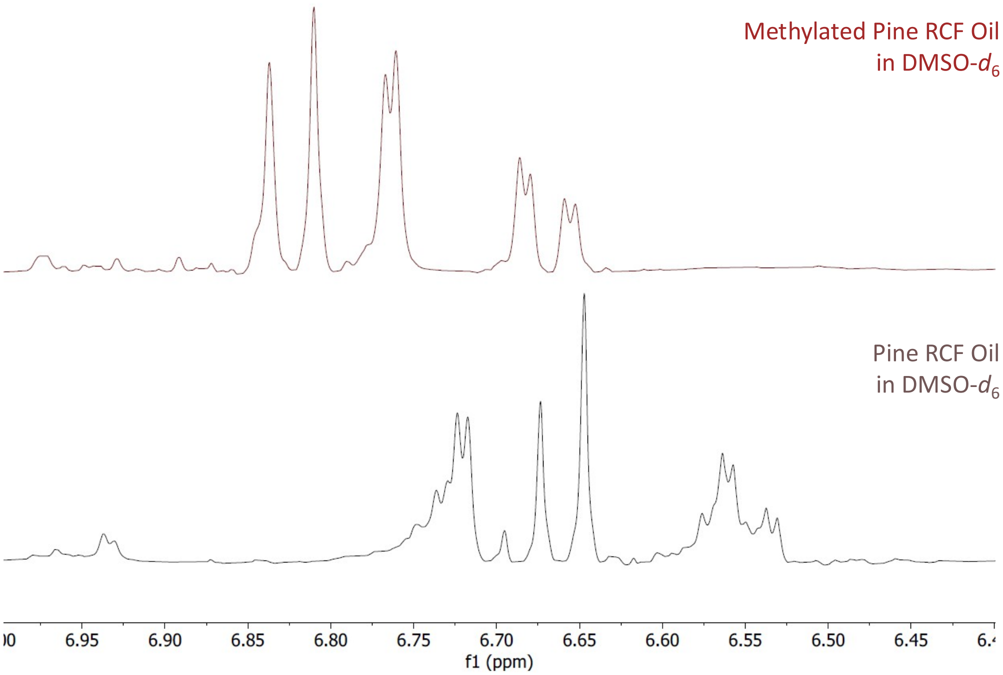

Supplementary Fig. 30. ^{1}H NMR spectra comparing the aromatic region pine RCF oil (bottom) and methylated pine RCF oil in DMSO-d_6.

Methylation of poplar RCF oil. A 250 mL round-bottom flask was charged with poplar RCF oil (7.547 g), potassium carbonate (30.0 g, 0.211 mol), acetonitrile (125 mL), and a stir-bar. Methyl iodide (61.0 g, 0.441 mol) was added, and the solution was stirred at room temperature. After 60 h, water (200 mL) was added to the resultant brown slurry, and the mixture was extracted with ethyl acetate (3 x 150 mL). The organic layers were combined and washed with water (2 x 100 mL) and brine (1 x 100 mL). The organic layer was subsequently dried with sodium sulphate, filtered, and the volatiles were removed by rotary evaporation leaving a brown viscous oil (9.131 g).

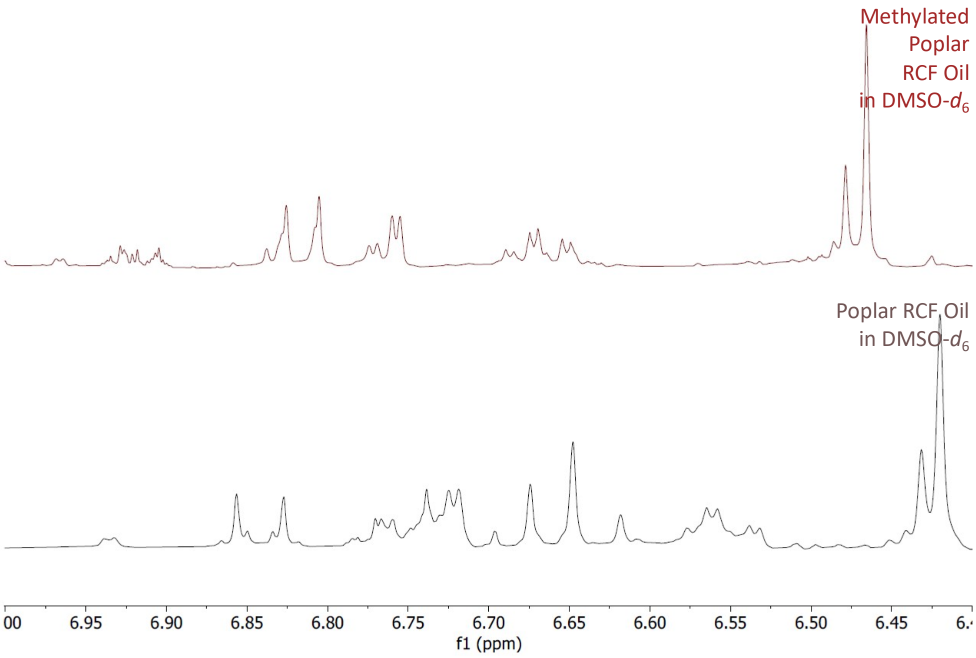

Supplementary Fig. 31. ^1H NMR spectra comparing the aromatic region poplar RCF oil (bottom) and methylated poplar RCF oil in DMSO-d_6.

Preparation of oligomer-enriched pine substrate. To a 25 mL round-bottom flask was added methylated pine RCF oil (7.001 g) and the mixture was placed in a Kugelrohr distillation apparatus with a collection flask submerged in a dry ice / acetone bath. The apparatus was heated to 220 °C at 10 mbar for 30 min affording an oligomer-enriched residue fraction along with a pale-yellow distillate (3.112g, 44 wt%). The residue was subsequently dissolved in EtOAc, filtered, and dried under vacuum. The resultant oil was triturated with methanol affording an oligomer-enriched substrate as a brown powder (2.794 g, 40 wt%). The total mass recovery was 84%.

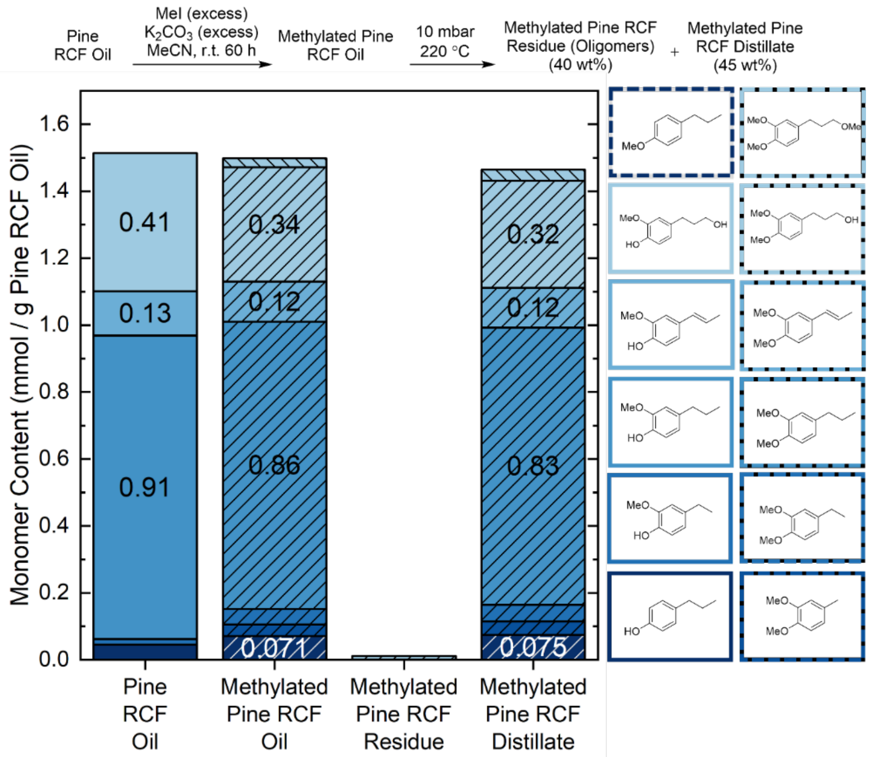

Supplementary Fig. 32. GC-FID quantification of monomers in the native pine RCF product, methylated pine RCF oil, the methylated pine distillation residue (oligomer substrate), and the methylated pine distillate showing quantitative recovery of all monomers.

Supplementary Table 3. GC-FID quantification of monomers in pine RCF oil.

	Analyte (mmol / g pine RCF oil)				
Substrate	4-Propyl Phenol	4-Ethyl Guaiacol	4-Propyl Guaiacol	Isoeugenol	4-Propanol Guaiacol
Pine RCF Oil	0.045	0.017	0.907	0.133	0.417

Supplementary Table 4. GC-FID quantification of monomers in whole and distilled methylated pine RCF oil.

	Analyte (mmol / g pine RCF oil)						
Substrate	4-Propyl Anisole	4-Methyl Veratrole	4-Ethyl Veratrole	4-Propyl Veratrole	Methyl Isoeugenol	3-Methoxy Propylveratrole	Propanol Veratrole
Methylated Pine RCF	0.071	0.035	0.047	0.858	0.119	0.028	0.342
Residue	0	0	0	8.2 x 10^{-4}	0	0	0.011
Distillate	0.075	0.040	0.049	0.829	0.118	0.032	0.321

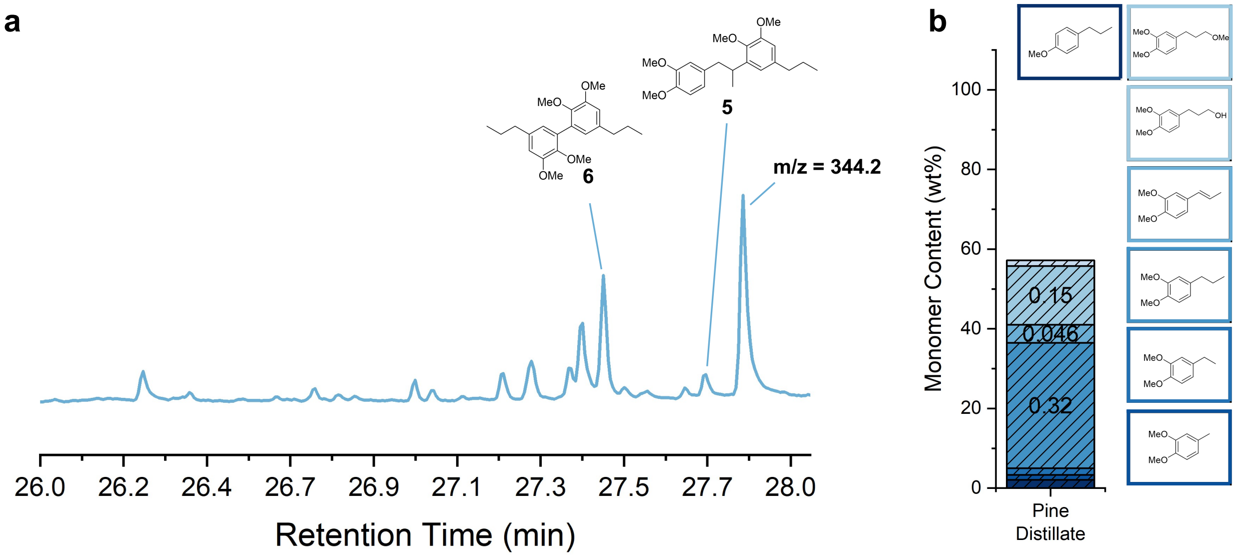

Supplementary Fig. 33. (a) GC-MS trace of the methylated pine distillate (blue) showing dimers **5** and **6** along with other compounds and **(b)** quantification of the monomers comprising 57 wt% of the distillate.

Preparation of Oligomer-enriched Poplar Substrate. To a 25 mL round-bottom flask was added methylated poplar RCF oil (7.914 g) and the mixture was placed in a Kugelrohr distillation apparatus with a collection flask submerged in a dry ice / acetone bath. The apparatus was heated to 220 °C at 10 mbar for 30 min affording an oligomer-enriched residue fraction along with a pale-yellow distillate (3.765 g, 48 wt%). The residue was subsequently dissolved in EtOAc, filtered, and dried under vacuum. The resultant oil was triturated with methanol affording an oligomer-enriched substrate as a brown powder (3.389 g, 43 wt%). The total mass recovery was 90%.

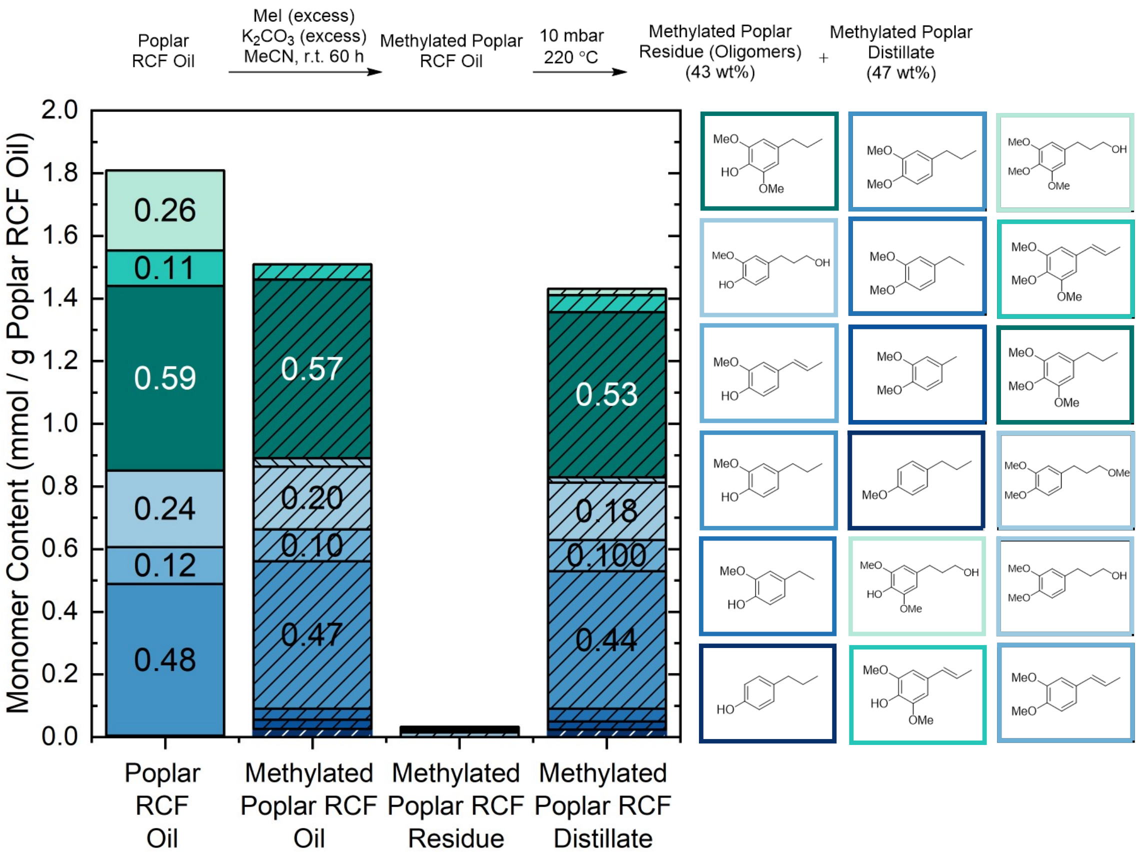

Supplementary Fig. 34. GC-FID quantification of monomers in the native poplar RCF product, methylated poplar RCF oil, the methylated poplar distillation residue (oligomer substrate), and the methylated poplar distillate.

Supplementary Table 5. GC-FID quantification of monomers in poplar RCF oil.

	Analyte (mmol / g poplar RCF oil)						
Substrate	4-Ethyl Guaiacol	4-Propyl Guaiacol	Isoeugenol	4-Propyl Syringol	4-Propanol Guaiacol	isoelemicin	4-Propanol Syringol
Poplar RCF Oil	0.005	0.484	0.119	0.590	0.244	0.113	0.255

Supplementary Table 6. GC-FID quantification of monomers in whole and distilled methylated poplar RCF oil.

	Analyte (mmol / g poplar RCF oil)					
Substrate	4-Propyl Anisole	4-Methyl Veratrole	4-Ethyl Veratrole	4-Propyl Veratrole	Methyl Isoeugenol	1-(3,4,5-trimethoxyphenyl) propane
Methylated Poplar RCF	0.26	0.030	0.035	0.470	0.101	0.570
Residue	0	0	0	6.9×10^{-4}	0	4.5×10^{-4}
Distillate	0.024	0.026	0.040	0.439	0.100	0.528

Supplementary Table 7. GC-FID quantification of monomers in whole and distilled methylated poplar RCF oil (continued).

	Analyte (mmol / g poplar RCF oil)				
Substrate	3-Methoxy Propylveratrole	Isoelemicin	4-Propanol Veratrole	3-Methoxy-(3',4',5'-trimethoxyphenyl) propane	4-Propanol (3',4',5'-trimethoxyphenyl) propane

Methylated Poplar RCF	0.027	0.043	0.200	0	0
Residue	0	0.006	0.014	0.003	0.008
Distillate	0.017	0.048	0.183	0	0.021

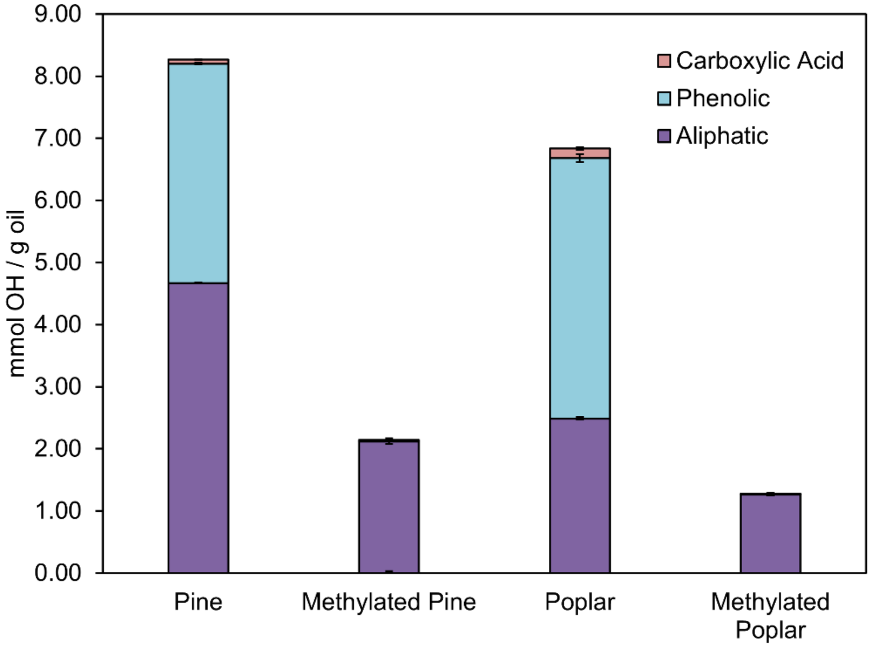

Supplementary Fig. 35. ^{31}P NMR quantification of aliphatic and phenolic hydroxyl groups (mmol / g sample) for the RCF oils and methylated RCF oils

.

Supplementary Table 8. ^{31}P NMR quantification of aliphatic and phenolic hydroxyl groups (mmol / g sample) for the RCF oils and methylated RCF oils.

Sample	Aliphatic	Phenolic	Carboxylic Acid	Total
Pine	4.67	3.53	0.07	8.27
Methylated Pine	2.12	0	0.02	2.14
Poplar	2.49	4.19	0.15	6.84
Methylated Poplar	1.26	0	0.02	1.28

Autoxidation Catalysis of the Oligomer Fractions

For a single reaction, a 75 mL Parr batch reactor fit with a glass liner insert was charged with 20-65 mg of functionalized RCF oil, acetic acid (15 mL), a stir bar, and $Mn(OAc)_2$•$4H_2O$ and $Zr(acac)_4$ catalyst. The mixture was pressurized three times with pure nitrogen and subsequently charged with air and nitrogen to achieve the desired oxygen partial pressure. The vessel was heated to temperature, at which, it was maintained for the desired timeframe before it was cooled rapidly in an ice bath. The solutions were stored in the freezer until needed for analysis. The methylated pine oligomers oxidation was run in triplicate. The methylated poplar oligomers oxidation was run in duplicate.

Supplementary Table 9. Numerical values for the GC-FID quantification of the products (mmol / g oligomer substrate) from the temperature screening study of the methylated pine RCF oligomer oxidation. MMA = 2-methoxymaleic acid, AA = anisic acid, VA1 = veratraldehyde, VA2 = veratric acid, DMI = dimethoxyisophthalate, DD1 = dimethyl divanillin, MDV = 2,2',3,3'-Tetramethoxybiphenyl-1'formyl-1-carboxylic acid, and DD2 = dimethyl divanillate.

	Analyte (mmol / g oligomer substrate)								
Temp (°)	MMA	AA	VA1	VA2	DMI	DD1	MDV	DD2	Total
90	0	0	0	0	0	0	0	0	0
110	0	0	0.011	0	0	0	0	0	0.011
130	0	0	0.176	0.043	0.008	0	0	0	0.227
150	0.093	0.280	0.331	0.544	0.043	0.027	0.068	0.041	1.18
170	0.069	0.031	0.232	0.557	0.049	0.019	0.054	0.043	1.06
190	0.062	0.038	0.076	0.527	0.057	0.007	0.031	0.041	0.840

Supplementary Table 10. Numerical values for the GC-FID quantification of the products (mmol / g oligomer substrate) from the oxygen loading study of the methylated pine RCF oligomer oxidation. MMA = 2-methoxymaleic acid, AA = anisic acid, VA1 = veratraldehyde, VA2 = veratric acid, DMI = dimethoxyisophthalate, DD1 = dimethyl divanillin, MDV = 2,2',3,3'-Tetramethoxybiphenyl-1'formyl-1-carboxylic acid, and DD2 = dimethyl divanillate.

	Analyte (mmol / g oligomer substrate)								
O_2 (bar)	MMA	AA	VA1	VA2	DMI	DD1	MDV	DD2	Total
0	0	0	0	0	0	0	0	0	0
1	0.057	0.021	0.298	0.365	0.026	0.022	0.047	0.025	0.860
2	0.057	0.023	0.339	0.427	0.030	0.025	0.067	0.031	0.999
4	0.090	0.031	0.261	0.579	0.051	0.017	0.053	0.046	1.13
6	0.093	0.028	0.331	0.544	0.043	0.027	0.068	0.041	1.18
8	0.102	0.034	0.170	0.583	0.056	0.011	0.036	0.041	1.03

Supplementary Table 11. Numerical values for the GC-FID quantification of the products (mmol / g oligomer substrate) from the catalyst loading study of the methylated pine RCF oligomer oxidation. MMA = 2-methoxymaleic acid, AA = anisic acid, VA1 = veratraldehyde, VA2 = veratric acid, DMI = dimethoxyisophthalate, DD1 = dimethyl divanillin, MDV = 2,2',3,3'-Tetramethoxybiphenyl-1'formyl-1-carboxylic acid, and DD2 = dimethyl divanillate.

	Analyte (mmol / g oligomer substrate)								
Cat. Loading Mn/Zr (wt%)	MMA	AA	VA1	VA2	DMI	DD1	MDV	DD2	Total
0/0	0	0	0.027	0.005	0	0	0	0	0.032
1/1.5	0	0.009	0.054	0.016	0	0	0	0	0.078
2/3	0	0.009	0.054	0.021	0	0	0	0	0.084
4/6	0.013	0.010	0.079	0.048	0.007	0	0	0.005	0.161
8/12	0.085	0.025	0.312	0.448	0.037	0.017	0.043	0.029	0.995
12/18	0.079	0.026	0.351	0.480	0.037	0.018	0.046	0.030	1.07
20/30	0.059	0.026	0.359	0.462	0.040	0.020	0.045	0.028	1.04

Supplementary Table 12. Numerical values for the GC-FID quantification of the products (mmol / g oligomer substrate) from the substrate loading study of the methylated pine RCF oligomer oxidation. MMA = 2-methoxymaleic acid, AA = anisic acid, VA1 = veratraldehyde, VA2 = veratric acid, DMI = dimethoxyisophthalate, DD1 = dimethyl divanillin, MDV = 2,2',3,3'-Tetramethoxybiphenyl-1'formyl-1-carboxylic acid, and DD2 = dimethyl divanillate.

	Analyte (mmol / g oligomer substrate)								
Concen-tration (mg/mL)	MMA	AA	VA1	VA2	DMI	DD1	MDV	DD2	Total
1.3	0.075	0.026	0.227	0.238	0.034	0.015	0.018	0.011	0.626
2.7	0.074	0.025	0.282	0.427	0.029	0.011	0.026	0.019	0.867
4.3	0.083	0.026	0.241	0.496	0.036	0.009	0.025	0.027	0.917

Supplementary Table 13. Numerical values for the GC-FID quantification of the products (mmol / g oligomer substrate) from time course study of the methylated pine RCF oligomer oxidation. MMA = 2-methoxymaleic acid, AA = anisic acid, VA1 = veratraldehyde, VA2 = veratric acid, DMI = dimethoxyisophthalate, DD1 = dimethyl divanillin, MDV = 2,2',3,3'-Tetramethoxybiphenyl-1'formyl-1-carboxylic acid, and DD2 = dimethyl divanillate.

	Analyte (mmol / g oligomer substrate)								
Time (h)	MMA	AA	VA1	VA2	DMI	DD1	MDV	DD2	Total
0.167	0.030	0	0.376	0.232	0.014	0.019	0.030	0.010	0.711
0.5	0.060	0.020	0.388	0.362	0.022	0.020	0.034	0.019	0.924
1	0.068	0.021	0.343	0.389	0.027	0.016	0.032	0.018	0.914
1.5	0.101	0.033	0.212	0.603	0.049	0	0.043	0.045	1.09
3	0.115	0.041	0.034	0.558	0.071	0.005	0.011	0.030	0.865
6	0.100	0.039	0.023	0.569	0.067	0.005	0.009	0.027	0.839

Supplementary Table 14. Numerical values for the GC-FID quantification of the products (mmol / g oligomer substrate) of the methylated pine RCF oligomer oxidation. MMA = 2-methoxymaleic acid, AA = anisic acid, VA1 = veratraldehyde, VA2 = veratric acid, DMI = dimethoxyisophthalate, DD1 = dimethyl divanillin, MDV = 2,2',3,3'-Tetramethoxybiphenyl-1'formyl-1-carboxylic acid, and DD2 = dimethyl divanillate.

	Analyte (mmol / g oligomer substrate)								
Zr Loading (wt%)	MMA	AA	VA1	VA2	DMI	DD1	MDV	DD2	Total
0	0	0	0.042	0.019	0	0	0.005	0	0.061
6	0.053	0.018	0.311	0.333	0.024	0.012	0.020	0.015	0.765
12	0.065	0.024	0.283	0.462	0.032	0.011	0.024	0.023	0.901
24	0.052	0.021	0.321	0.374	0.026	0.012	0.021	0.016	0.821

Supplementary Table 15. Numerical values for the GC-FID quantification of the products (mmol / g oligomer substrate) of the methylated pine RCF oligomer oxidation. MMA = 2-methoxymaleic acid, AA = anisic acid, VA1 = veratraldehyde, VA2 = veratric acid, DMI = dimethoxyisophthalate, DD1 = dimethyl divanillin, MDV = 2,2',3,3'-Tetramethoxybiphenyl-1'formyl-1-carboxylic acid, and DD2 = dimethyl divanillate.

	Analyte (mmol / g oligomer substrate)								
	MMA	AA	VA1	VA2	DMI	DD1	MDV	DD2	Total
Run 1	0.089	0.038	0.123	0.665	0.059	0.007	0.022	0.039	1.04
Run 2	0.122	0.036	0.108	0.614	0.063	0.006	0.042	0.039	1.03
Run 3	0.064	0.023	0.322	0.454	0.029	0.012	0.029	0.021	0.95
Avg	0.092	0.032	0.184	0.578	0.050	0.008	0.031	0.033	1.01

Supplementary Table 16. Numerical values for the GC-FID quantification of the products of the methylated poplar RCF oligomer oxidation. The oxidations were run in duplicate. MMA = 2-methoxymaleic acid, AA = anisic acid, VA1 = veratraldehyde, DMBQ = 2,6-dimethoxybenzoquinone, VA2 = veratric acid, TMB = 3,4,5-trimethoxybenzaldehyde, TMBA = 3,4,5-trimethoxybenzoic acid, DMI = dimethoxyisophthalate, DD1 = dimethyl divanillin, MDV = 2,2',3,3'-Tetramethoxybiphenyl-1'formyl-1-carboxylic acid, and DD2 = dimethyl divanillate.

	Analyte (mmol / g oligomer substrate)											
	MMA	AA	VA1	DMBQ	VA2	TMB	TMBA	DMI	DD1	MDV	DD2	Total
Run 1	0.050	0.030	0.120	0.166	0.114	0.192	0.245	0.016	0.018	0.018	0.012	0.981
Run 2	0.044	0.031	0.130	0.161	0.127	0.182	0.241	0.008	0.003	0.005	0.002	0.934
Avg	0.047	0.031	0.125	0.164	0.121	0.187	0.243	0.012	0.011	0.012	0.007	0.958

Biological Conversion

Strain construction

Specific plasmid and strain construction methods are provided in **Supplementary Table S17** and **Supplementary Table S20**, respectively. Primer and synthetic gene sequences are provided in **Supplementary Table S18** and **Supplementary Table S19**, respectively. PCR reactions were carried out with Q5® High-Fidelity 2X Master Mix (New England Biolabs). For plasmid-based expression of genes in *Pseudomonas putida* (hereafter *P. putida*), the pBTL-2 backbone (Genbank: DQ058740.1) underwent restriction enzyme digestion and genes of interest were cloned in by Gibson assembly according to manufacturer instructions (NEBuilder HiFi DNA Assembly Master Mix, New England Biolabs). Strains were prepared for electroporation according to a previously established method,[22] electroporated with ~100 ng of pBTL-2-based plasmid, and selected on LB agar with 50 μg/mL kanamycin. For genomic expression of genes in *P. putida*, flanking ~1 kb DNA fragments with homology to the desired genomic locus were assembled with fragment(s) for the gene of interest into the suicide vector pK18sB (Genbank: MH166772). Host strains were electroporated with pK18sB-based plasmids, followed by antibiotic selection and sucrose counter-selection according to a previously described protocol.[23]

Preparation of bacterial culture media

Oxidations of methylated pine RCF oligomers were performed in triplicate using the optimized conditions. Aliquots of each sample were added to a vial, yielding approximately 30 mg of the oxidation product mixture upon evaporation of the acetic acid solvent under a flow of N_2 at room temperature. A stir bar was added to each vial, and the dried material was solubilized by stirring with 2 mL of water and 2.2 mL of aqueous 4 M NaOH for 1 h at room temperature. The high pH of the solution precipitated the metal catalyst, and it was removed by centrifuging the samples at 3000 g for 5 min. Each supernatant was transferred to a 25 mL flask with a stir bar, and the solutions were neutralized by dropwise addition of 38% HCl. Preparations were then sterilized by passage through 0.2 μm syringe filters. For culture media, sterile RCF oxidation substrate was mixed with 1.1X M9 medium to achieve a final concentration of 10% v/v oxidized RCF stream in 1X M9 medium (6.78 g/L Na_2HPO_4, 3 g/L KH_2PO_4, 0.5 g/L NaCl, 1 g/L NH_4Cl, 2 mM $MgSO_4$, 100 μM $CaCl_2$, and 18 μM $FeSO_4$). The initial compositions of these media are listed in **Supplementary Table S10**. For control media, 20 mM stock solutions of veratrate (3,4-dimethoxybenzoic acid, Sigma) and veratraldehyde (3,4-dimethoxybenzaldehyde, Sigma) were prepared in water, dissolved with 4 M aqueous NaOH to neutral pH, and filter sterilized. These stocks were combined with 1.1X M9 medium to generate 1X M9 medium with 2 mM of either aromatic.

Shake flask cultivations

Three cultures of ACB263 were prepared in 5 mL LB medium (Sigma-Aldrich) in 15 mL glass culture tubes, using a single colony from an agar plate to inoculate each tube. A single culture of ACB262 was prepared in the same manner. Cultures were incubated overnight (~16 h) at 225 rpm and 30°C, and then each culture was centrifuged at 2500 *g* for 5 min to pellet the cells. Cell pellets were resuspended in 2 mL each of 1X M9 medium without carbon. Sterile media were added to 50 mL culture flasks, using 8 mL medium per flask. Cell suspensions were then transferred to the flasks to achieve an initial OD_{600nm} of 0.1. Three flasks per condition were inoculated with ACB263 (biological triplicates), one flask per condition was inoculated with ACB262, and one flask per condition remained uninoculated as an abiotic control. Sterile glucose solution was added to a final concentration of 5 mM for all flasks containing ACB263, and each flask was sealed with Breathe-Easy film (Diversified Biotech) and a foam stopper to limit evaporation. Flasks were incubated at 225 rpm and 30°C for 5 days. Periodically, volumes of 250-400 μL were withdrawn from each flask. 50 μL of each sample was used for OD_{600nm} measurement on a spectrophotometer (Mettler-Toledo UV5BIO), and the remaining volume was centrifuged at 6000 *g* for 1 min and filtered through a 0.2 μm membrane for metabolite analysis. An additional 5 mM glucose was added to flasks containing ACB263 every 24 h to support growth and conversion of the substrates.

Plasmids

Supplementary Table 17. Plasmids used in this study.

Plasmid	Utility	Construction details
**pBTL-2**	pBBR1-based broad host range plasmid used for expression in *P. putida* KT2440	GenBank: DQ058740.1 [24]
**pK18sB**	Sucrose counter-selection allelic exchange vector for *P. putida* KT2440	GenBank: MH166772.1 [25]
**pK18sB PP5322-PP5323**	pK18sB-based suicide vector for integration of genes at the PP_5322 locus in *P. putida* KT2440	Synthesized by TWIST Biosciences
**pACB086**	pK18sB-based suicide vector for integration of the CYP199A4-HaPux operon at the *fpvA* locus in *P. putida* KT2440	Synthesized by TWIST Biosciences
**pACB090**	pBTL-2-based replicative plasmid for expression of the CYP199A4-HaPux-HaPuR operon in *P. putida* KT2440	The HaPuR gene (RPB_RS18445) from *R. palustris* HaA2 was amplified from an IDT gBlock (HaPuR) with oACB369 and oACB370. The CYP199A4-HaPux operon was amplified from pACB086 with oACB371 and oACB372. The two products were assembled by the method of Gibson into pBTL-2 digested with XbaI & EcoRV. The plasmid was Sanger sequenced with primers oCJ162, oCJ479, oACB367, oACB350, oACB352, oACB369, and oCJ163.
**pACB098**	pK18sB-based suicide vector for integration of the CYP199A4-HaPux-HaPuR operon at the PP_5322 locus in *P. putida* KT2440	pK18sB_PP5322-PP5323 was amplified with oACB393 and oACB394. The CYP199A4-HaPux-HaPuR operon was amplified from pACB090 with oACB395 and oACB396. Primers introduced

		a TonB terminator after HaPuR, since this was absent in the pK18sB_PP5322-PP5323 backbone. The two products were assembled by the method of Gibson. The plasmid was Sanger sequenced with primers oACB397, oACB395, oACB367, oACB350, oACB369, and oACB398.
**pACB118**	pK18sB-based suicide vector for integration of the CYP199A4-HaPux-HaPuR operon at the PP_5322 locus in *P. putida* KT2440, including a mutation in the RBS for HaPux	pACB098 was amplified with site-directed mutagenesis primers oACB451 and oACB452 to make a linear 7504 bp product with the C>G mutation in the HaPux RBS. The product was re-ligated in a KLD reaction. The plasmid was Sanger sequenced with oACB397, oACB395, oACB367, oACB350, oACB369, and oACB398.
**pACB124**	pK18sB-based suicide vector for integration of the *ivaAB* operon at the PP_5042 locus in *P. putida* KT2440	Synthesized by TWIST Biosciences
**pCA004**	pK18sB-based suicide vector for creation of E474V mutation in *aroY* in *P. putida* strain CJ781	Synthesized by TWIST Biosciences

DNA Sequences

Supplementary Table 18. DNA Sequences of oligos used in this study. Integrated DNA Technologies (IDT) was used for synthesis.

Primer	Sequence (5'→3')	Description
**oACB350**	CGACCCAGACCTGTACGAC	Diagnostic: binds toward 3' end of CYP199A4. Forward.
**oACB352**	GCGGATAACAATTTCACACTCGCACCAGATTTA CATAATACCTAAG	Diagnostic: binds RBS of HaPux. Forward.
**oACB367**	GTGAGCACCTGCTGCCTTACG	Diagnostic: binds in middle of CYP199A4. Forward.
**oACB369**	GTCAGACCTGATCACAACAACATATAGGAG	Amplifies HaPuR for assembly into pACB0990. Forward.
**oACB370**	TCTGAGGCTCGTCCTGAATGATTTAAGCCATGG CTTTCTTC	Amplifies HaPuR for assembly into pACB090. Reverse.
**oACB371**	GAGCGGATAACAATTTCACACTAAATCAGTCCA TAACAGGAGATAACAATG	Amplifies CYP199A4-HaPux for assembly into pACB090. Forward.
**oACB372**	TGTTGTTGTGATCAGGTCTGACGTTCCGG	Amplifies CYP199A4-HaPux for assembly into pACB090. Reverse.
**oACB393**	CTCCGGTCGGAGGCTTTTGACTAATCGGTCAGG GGTTGCC	Amplifies pK18sB_PP5322-PP5323 for assembly into pACB098. Forward.
**oACB394**	GTGTGAAATTGTTATCCGCTCAC	Amplifies pK18sB_PP5322-PP5323 for assembly into pACB098. Forward.
**oACB395**	AGCGGATAACAATTTCACACAAATCAGTCCATA ACAGG	Amplifies CYP199A4-HaPux-HaPuR for assembly into pACB098. Forward.
**oACB396**	CAACCCCTGACCGATTAGTCAAAAGCCTCCGAC CGGAGGCTTTTGACTTTTAAGCCATGGCTTTCT TC	Amplifies CYP199A4-HaPux-HaPuR for assembly into pACB098. Adds 3' TonB terminator. Reverse.
**oACB397**	AAGACGTGCTGGAAGTGC	Diagnostic: binds in PP_5322 upstream targeting sequence in pACB098. Forward.
**oACB398**	CTCGGGTAACTTTGTCAGG	Diagnostic: binds in PP_5323 downstream targeting sequence in pACB098. Reverse.
**oACB399**	CTGTGCATTGCTCATGGC	Diagnostic: binds outside PP_5323 downstream targeting region in KT2440. Reverse.
**oACB451**	ATACCTAAG**G**AGTATAGTTATGCCGAGC	For site directed mutagenesis (C→G, underlined) of HaPux RBS in pACB118. Forward.
**oACB452**	TATGTAAATCTGGTGCGTCAAGC	For site directed mutagenesis of HaPux RBS in pACB118. Reverse.
**oACB466**	CAGGTTGCTCAGGCTGTCGTAC	Diagnostic: binds outside PP_5042 upstream targeting region in KT2440. Forward.
**oACB467**	GCATTGGCTGCGTAACGAAGC	Diagnostic: binds outside PP_5042 downstream targeting region in KT2440. Reverse.
**oACB468**	TTGGGCGTTGAGTCTTGC	Diagnostic: binds upstream of Ptac in pACB124 Forward.
**oACB469**	CAGTTCCGTAACCAGCAGC	Diagnostic: binds in 3' end of *ivaA* in pACB124. Forward.
**oACB470**	GTGGGTTCAGTGCTTCATGC	Diagnostic: binds downstream of TonB terminator in pACB124. Reverse.
**oCJ054**	ATCGGCTCGTATAATGTGTGG	Diagnostic: binds in Ptac promoter. Forward.
**oCJ162**	CCCAGGCTTTACACTTTATGC	Diagnostic: binds in lac promoter in pBTL-2. Forward.
**oCJ163**	TTGTCCAGCAGGGTTGTC	Diagnostic: binds downstream of insert sequence in pBTL-2. Reverse.

**oCJ479**	GTTTCACTTGATGCTCGATG	Diagnostic: binds upstream of lac promoter in pBTL-2. Forward.

Synthetic Genes

Supplementary Table 19. Synthetic genes used in this study. TWIST Biosciences & Integrated DNA Technologies (IDT) were used for synthesis.

Name	Sequence (5'→3')	Description
ivaAB	GAGCTGTTGACAATTAATCATCGGCTCGTATAATGTGTGGAATTGTGAGCGGATAACAAT<br>TTCACACGTTATTTCCCTTAAGGAACCGTTAATGAACCAGATCTCCCACACCATGAGCGC<br>CGCGGCACCAGCGTTCCCGCTCGACCAGTGGTACGTTGCGGGTTTCGCCTGGGAGCTGAA<br>AGACCTGCCGGTTGCTCGCACCCTGCTGGGTCAGCCGGTTGTTCTGTTCCGCACCGAAGA<br>CGGCCGCGTTGCCGCCCTGGAAGACCGCTGTTGCCACCGCGAACTGCCGCTCAGTTGCGG<br>CACTGTGGAAGCTGCGGGTCTGCGCTGCGGCTACCACGGTCTGCTGTTCTCGCACGAAGG<br>TCAATGCCTGGAAATCCCGGGTCAGGAACGAATCCCAACCAAAGCCTGCGTTAAGTCCTT<br>CGAACTGCGCGAACGTGACCAAATCCTGTGGATCTGGATGGGTGCTACCCCGGACAGCGT<br>GCCAGGTACCGAGCCACCGGCCTACGCTGTGCACTCCGACCCGCAGTACCGCTTCGGCGG<br>CGGCGTCTACCACTACGACGCCCCATACCAGCTGATCCACGACAACCTGCTGGACCTGTC<br>CCACCTGGGCTACGTACACCTGAAGACTATTGGCGGCAACGCGCGCGTCCACATGAACGC<br>TGACCTGAAGGTCTCCCAGGACGGTGACAGCGTCAAGGTTGTGCGTTGGATGCCGGACTC<br>CGACCCACCGCCTACTTACGCCCAGGCATGGCCTTTCCAGGGCCGAATCGATCGTTGGCA<br>GGAAGTTGAATTCCACCCATCCCACGTGCGCATCTGGACCGGCGCCATGGACGCCGGCCA<br>GGACCGCCTGGACAACCCAGCTCGTGAGGGCTTCCACATGCGTGGCTTCCACGGCGTCAC<br>CCCTGAAACCGAGACCAGCGCACACTACTTCTGGACCATCGCCACCAACCCTCACCCACA<br>GATGCAGGACACCACCCAACTCGTAATCGACCAAACCGCCGCAACCTTCGAGGAAGACAA<br>GGTTGTCATTGAGGCTCAGTTCCGTAACCAGCAGCGCTTCGGCCCACGTGCCGTCGTTGA<br>CATCCACGTTGACGTGGGCCCGAACCGTGCACGTCGCGTTATCGAACGTCTGCGCCAGGC<br>CGGCGCCCAAGCTAGCCAGGCCGTAGCCTAGAGGGAAATCAAGACAAGCAGGGGGGTTTA<br>TATGAAGACCGAGACGACCTTCGAAGTTCGCATCGCGCACAAGCAGGAGCAGGCCGCCGG<br>TATCTGCTCGCTGGAGCTGCGTGCACTGGAAGGCGCTTCCCTGGCTCCCTTCTCCGCCGG<br>CAGTCACATCGACGTCCACCTGCCAGGTGGTCTGGTCCGCCAATACTCGCTGTCGAACGA<br>CCCGGCGGAACTGGACCGCTACGTGATCGCCGTCCTGCGTGAACCGGCGTCCCGTGGTGG<br>TTCGGCCGCAGTCCACGAACAGCTGCAGGCTGGCCAGCAGATCACCATTTCCCACCCTCG<br>CAACCACTTCGAACTGCACGCCCCAGCTCGCAAGCACCTGCTGCTGGCTGGCGGCATCGG<br>TATTACCCCGATCCTCGCGATGGCCCGCAAGCTGGCCCACGACGGTGCCGAGTTCGCACT<br>GCATTACTGCGGCCGCTCGCGTGAACGTATGGCTTTCGCCCAGACCATCGAAACCGCGCA<br>ATGGGCTGACAAGGCGCAGATCCACGTCGACGACGTCAACGGTAAGTCGTCGCTGGGCCT<br>GTCCGAGCTGCTGCAGCAGCGCGAGCAGGGCCAACATCTGTACGTGTGCGGCCCAAAGGG<br>TTTCATGGACGCCGTGCTGGACACCGCACGCGCCGCTGGTTGGCCTGCCGAGCAGCTGCA<br>CTACGAATTCTTCGCCGCCGAGGTGGAGCACCGTGACGATGACGAGAGCTTCGAGGTCGA<br>GGTTGCATCCTCGGGCCAAGTGGTTCGCGTCACCCCAGCGCAGACCGTTGTGCAGGCTCT<br>GGAATCCATCGGCGTATGCGTCCAGACCAGCTGCGAGCAGGGCGTTTGCGGCACGTGCCT<br>GACCCGTGTTATCTCGGGCGAACCAGACCACCGCGACATGTACTTGACCGAAGACGAGCA<br>GGCCGCCAACGATCAGTTCCTGCCATGCTGCTCCCGTGCTCGTTCGGGCCGCCTGGTGCT<br>GGACCTGTGAAGTCAAAAGCCTCCGACCGGAGGCTTTTGACT	From TWIST Biosciences. The *ivaA* (gray) and *ivaB* sequences (green) from *C. testosteroni* BR6020 were codon optimized for expression in *P. putida* KT2440. The construct also includes the P_{tac} promoter (blue), synthetic ribosome binding sites (red), and TonB terminator (purple).
**CYP199A4-HaPux**	GAGCTGTTGACAATTAATCATCGGCTCGTATAATGTGTGGAATTGTGAGCGGATAACAAT<br>TTCACACAAATCAGTCCATAACAGGAGATAACAATGATCAGCAACTCCAGCGCCGAATCG<br>ATCTCGGCTCCGCCTAACGACTCGACCATCCCGCACCTGGCCATCGACCCGTTCAGCCTG<br>GACTTCTTCGACGACCCATACCCGGACCAACAAACCCTGCGTGACGCAGGTCCAGTTGTG<br>TACCTGGACAAATGGAACGTGTACGGTGTAGCCCGCTACGCCGAAGTTCACGCTGTGCTG<br>AACGACCCAACCACCTTCTGCTCGAGCCGCGGTGTGGGCCTGTCCGATTTCAAAAAGGAA<br>AAGCCTTGGCGTCCGCCTTCCTTGATCCTTGAAGCCGATCCGCCTGCCCACACCCGCCCG<br>CGCGCTGTCCTGTCCAAGGTCCTGTCGCCGGCTACCATGAAAACCATCCGTGACGGCTTC<br>GCCGCGGCCGCCGACGCAAAGGTTGACGAGCTGCTGCAGCGTGGCTGTATCGATGCCATC<br>GCTGACCTGGCTGAAGCCTACCCACTGAGCGTGTTCCCGGACGCCATGGGTCTGAAGCAG<br>GAGGGTCGTGAGCACCTGCTGCCTTACGCCGGCCTGGTCTTCAACGCCTTCGGTCCGCCG<br>AACGAACTGCGCCAAACCGCCATCGAACGCTCCGCTCCACACCAGGCCTACGTGAACGAA<br>CAGTGCCAGCGCCCTAACTTGGCCCCGGGCGGCTTCGGCGCTTGCATCCACGCATTCACT<br>GACACCGGTGAGATCACCCCGGATGAGGCCCCACTTCTGGTCCGCTCGCTGCTGTCCGCT<br>GGCCTGGACACCACCGTCAACGGCATCGGCGCCGCAGTATACTGCCTGGCTCGTTTCCCG<br>GGCGAGTTACAGCGTCTGCGTAGCGACCCGACTCTGGCTCGCAACGCCTTCGAAGAAGCC<br>GTCCGCTTCGAAAGTCCGGTCCAGACCTTCTTCCGCACCACCACCCGCGAAGTGGAGCTG<br>GGCGGTGCCGTCATCGGCGAAGGTGAAAAAGTACTGATGTTCCTGGGCAGCGCCAACCGT<br>GACCCACGCCGCTGGTCCGACCCAGACCTGTACGACATCACCCGTAAGACTTCCGGTCAC<br>GTCGGCTTCGGCTCGGGCGTTCACATGTGCGTCGGCCAGCTCGTGGCCCGTCTGGAGGGC<br>GAAGTGATGCTGTCTGCCCTCGCTCGCAAAGTCGCTGCAATTGACATCGACGGCCCGGTC<br>AAACGTCGTTTCAACAACACCCTGCGCGGCCTGGAATCGCTGCCGGTAAAGCTGACCCCT<br>GCTTGACGCACCAGATTTACATAATACCTAAGCAGTATAGTTATGCCGAGCATTACCTTC<br>ATCCACCCGGACGGTCGCTCCGAAATTGTTGACGCCGCCATCGGCGACTCCGCTATGTTC<br>GCAGCGCTGAACCACGGTATCGACTCCATCGTTGCAGAGTGCGGTGGCAACGCCGTGTGC<br>GCCACCTGCCACGTCTACGTAGACGACCTCTGGCTTGCGAAGCTGCCGCCGGTGGACGCG	From TWIST Biosciences. The CYP199A4 (RPB_RS18230) sequence (gray) and HaPux (RPB_RS18235) sequence (green) from *R. palustris* HaA2 were codon optimized for expression in *P. putida* KT2440. The construct also includes the P_{tac} promoter (blue), synthetic ribosome binding sites (red), and TonB terminator (purple).

	AACGAAGACGACCTGTTGGACGGCACCGCTTCGGACCGCCTGCCTAACAGCCGCCTGTCC<br>TGCCAGATCAAGATCGCGCCAGAACTGGATGGTCTGGTTCTGCGCCTGCCGGAACGTCAG<br>ACCTGAAGTCAAAAGCCTCCGACCGGAGGCTTTTGACT	
**HaPuR**	TCACAACAACATATAGGAGGTAAGCATATATGAACGACACCGTCCTGATTGCCGGTGCCG<br>GCCATGCCGGCTTCCAGGTGGCGGTCAGCCTGCGTCAGGCTAAGTACACCGGTCGTATCG<br>CACTGATCAACGACGAAAAACACTTGCCGTACCAGCGCCCTCCGCTGAGCAAGGCCTACC<br>TGAAATCGGGTGGTGACCCGAACTCTCTGATGTTCCGTCCGGAAAAGTTCTTCCAAGATC<br>AGACCATCGAGCTGATCGACGGTCGCGCTGTCGCTATCGACCGCGACGCCAAGACCCTGC<br>TGCTCGCCTCGGGCGACAAGATCGAGTACGGCCACCTGGTACTGGCTACCGGTGCCCGCA<br>ACCGCCAGCTGGACGTCCCGAACGCCACCCTGGACGACGTGCTGTACCTGCGCACCTTGG<br>ATGAATCCGAGGTTGTGCGCCAGCGTATGCCGGAGAAGAAGCATGTTGTCGTGATCGGCG<br>CCGGCTTCATCGGCCTGGAGTTCGCTGCCACGGCTCGTGGTAAAGGCATGGAGGTCGACG<br>TCGTGGAACTGGCCCCGCGTGTGATGGCCCGTGCTGTCACCCCGGAGATCAGCTCGTACT<br>TCCATGATCGCCACACCGCGGCCGGCATCCGCATCCATTACGGCGTCCGTGCCACCGAGA<br>TCGAAGGCGAAGACGGCCGTGTCACCGGCGTTGCTCTCAGCGACGGCCGCACCCTGCCGT<br>GCGACCTGGTCGTGGTAGGCGTTGGTGTCATCCCCAACGTCGAACTGGCATCGGCCGCGG<br>GTCTGCCGACCGCAGCCGGCATCATCGTCAACGAACAGCTGCTGACCGAAGACCCGAACA<br>TCTCGGCCATCGGTGACTGCGCTCTGTTCAACTCGGTGCGTTTCGGCGAGGTCATGCGTG<br>TGGAGTCGGTCCAAAACGCAACCGACCAGGCCCGCTGCGTAGCCGCTCGTCTGACCGGCT<br>CGCCGGCTACCTACGACGGCTACCCCTGGTTCTGGAGCGACCAGGGTGATGACAAACTGC<br>AGATCGCCGGTCTGACCGCAGGCTTCGACCAGGTGGTTCTGCGTGGTTCCGTGGCGGAAC<br>GTAGTTTCAGCGCTTTCTGTTACAAGGACGGCCAGCTGATCGGCGTTGAGTCCGTGAACC<br>GCGCTGCGGATCACGTTTTCGGCCGTAAAATCCTCCCGCTGGGTAAGAGCGTTACCCCAG<br>AACAGGCTGCCGACCTGTCGTTCGACCTGAAGAAAGCCATGGCTTAA	From IDT. The HaPuR (RPB_RS18445) sequence (green) from *R. palustris* HaA2 was codon optimized for expression in *P. putida* KT2440. The construct also includes a synthetic ribosome binding site (red).

Strains and Construction Details for Bacterial Strains

Supplementary Table 20. Strains and construction details for bacterial strains used in this study.

Strain	Genotype	Construction details
**CA011**	*E. coli* DH5α F'Iq	Cloning strain; harbors pCA004
**ACB148**	*E. coli* DH5α F'Iq	Cloning strain; harbors pACB086
**ACB163**	*E. coli* DH5α F'Iq	Cloning strain; harbors pACB090
**ACB188**	*E. coli* DH5α F'Iq	Cloning strain; harbors pACB098
**ACB199**	*E. coli* DH5α F'Iq	Cloning strain; harbors pACB118
**ACB261**	*E. coli* DH5α F'Iq	Cloning strain; harbors pACB124
**CJ781**	*P. putida* KT2440 Δ*catRBCA*::P_{tac}:*catA* Δ*pcaHG*::P_{tac}:*aroY*:*ecdBD* Δ*crc* Δ*pobAR* Δ*fpvA*::P_{tac}:*praI*:*vanAB*	Previously described [26]
**CA022**	*P. putida* KT2440 Δ*catRBCA*::P_{tac}:*catA* Δ*pcaHG*::P_{tac}:*aroY*$_{E474V}$: *ecdBD* Δ*crc* Δ*pobAR* Δ*fpvA*::P_{tac}:*praI*:*vanAB*	CJ781 electrocompetent cells were transformed with ~500 ng of pCA004. Colonies were selected twice on LB + 50 mg/L kanamycin agar and twice on YT + 25% sucrose agar. Sequencing of the counterselected colonies verified the presence of the AroY(E474V) mutation in the genome.
**ACB164**	*P. putida* KT2440 + pACB090	*P. putida* KT2440 wild-type electrocompetent cells were transformed with ~100 ng of pACB090. Colonies were selected on LB + 50 mg/L kanamycin agar.
**ACB177**	CA022 + pACB090	CA022 electrocompetent cells were transformed with ~100 ng of pACB090. Colonies were selected on LB + 50 mg/L kanamycin agar.
**ACB180**	*P. putida* KT2440 Δ*catRBCA*::P_{tac}:*catA* Δ*pcaHG*::P_{tac}:*aroY*$_{E474V}$: *ecdBD* Δ*crc* Δ*pobAR* Δ*fpvA*::P_{tac}:*praI*:*vanAB* ΔPP_5322::P_{tac}:CYP199A4 -HaPux-HaPuR	CA022 electrocompetent cells were transformed with ~500 ng of pACB098. Colonies were selected twice on LB + 50 mg/L kanamycin agar and twice on YT + 25% sucrose agar. The counterselected colonies then underwent colony PCR with oACB397 and oACB399 to verify insertion of the CYP199A4-HaPux-HaPuR operon at the PP_5322 site. A colony with the correct 4157 bp insertion band was amplified again with oACB397 and oACB398, and the purified PCR product was sequenced with oACB397, oCJ054, oACB367, oACB350, oACB352, and oACB396 to verify the genomic sequence.
**ACB189**	*P. putida* KT2440 ΔPP_5322::P_{tac}:CYP199A4 -HaPux-HaPuR	*P. putida* KT2440 wild-type electrocompetent cells were transformed with ~500 ng of pACB098. Colonies were selected twice on LB + 50 mg/L kanamycin agar and twice on YT + 25% sucrose agar. The counterselected colonies then underwent colony PCR with oACB397 and oACB399 to verify insertion of the CYP199A4-HaPux-HaPuR operon at the PP_5322 site. A colony with the correct 4157 bp insertion band was amplified again with oACB397 and oACB398, and the purified PCR product was sequenced with oACB397, oCJ054, oACB367, oACB350, oACB352, and oACB396 to verify the genomic sequence.

**ACB236**	*P. putida* KT2440 ΔPP_5322::P_{tac}:CYP199A4-HaPux$_{newRBS}$-HaPuR	*P. putida* KT2440 wild-type electrocompetent cells were transformed with ~500 ng of pACB118. Colonies were selected twice on LB + 50 mg/L kanamycin agar and twice on YT + 25% sucrose agar. The counterselected colonies then underwent colony PCR with oACB397 and oACB399 to verify insertion of the CYP199A4-HaPux-HaPuR operon at the PP_5322 site. A colony with the correct 4157 bp insertion band was amplified again with oACB397 and oACB398, and the purified PCR product was sequenced with oACB397, oCJ054, oACB367, oACB350, oACB352, and oACB396 to verify the genomic sequence, including the C→G mutation in the HaPux RBS.
**ACB237**	*P. putida* KT2440 Δ*catRBCA*::P_{tac}:*catA* Δ*pcaHG*::P_{tac}:*aroY*$_{E474V}$:*ecdBD* Δ*crc* Δ*pobAR* Δ*fpvA*::P_{tac}:*praI*:*vanAB* ΔPP_5322::P_{tac}:CYP199A4-HaPux$_{newRBS}$-HaPuR	CA022 electrocompetent cells were transformed with ~500 ng of pACB118. Colonies were selected twice on LB + 50 mg/L kanamycin agar and twice on YT + 25% sucrose agar. The counterselected colonies then underwent colony PCR with oACB397 and oACB399 to verify insertion of the CYP199A4-HaPux-HaPuR operon at the PP_5322 site. A colony with the correct 4157 bp insertion band was amplified again with oACB397 and oACB398, and the purified PCR product was sequenced with oACB397, oCJ054, oACB367, oACB350, oACB352, and oACB396 to verify the genomic sequence, including the C→G mutation in the HaPux RBS.
**ACB262**	*P. putida* KT2440 ΔPP_5322::P_{tac}:CYP199A4-HaPux$_{newRBS}$-HaPuR ΔPP_5042::P_{tac}:*ivaAB*	ACB236 electrocompetent cells were transformed with ~500 ng of pACB124. Colonies were selected twice on LB + 50 mg/L kanamycin agar and twice on YT + 25% sucrose agar. The counterselected colonies then underwent colony PCR with oACB466 and oACB467 to verify insertion of the *ivaAB* operon at the PP_5042 site. A colony with the correct 4090 bp insertion band was amplified again with oACB466 and oACB467, and the purified PCR product was sequenced with oACB468, oACB469, and oACB470 to verify the genomic sequence.
**ACB263**	*P. putida* KT2440 Δ*catRBCA*::P_{tac}:*catA* Δ*pcaHG*::P_{tac}:*aroY*$_{E474V}$:*ecdBD* Δ*crc* Δ*pobAR* Δ*fpvA*::P_{tac}:*praI*:*vanAB* ΔPP_5322::P_{tac}:CYP199A4-HaPux$_{newRBS}$-HaPuR ΔPP_5042::P_{tac}:*ivaAB*	ACB237 electrocompetent cells were transformed with ~500 ng of pACB124. Colonies were selected twice on LB + 50 mg/L kanamycin agar and twice on YT + 25% sucrose agar. The counterselected colonies then underwent colony PCR with oACB466 and oACB467 to verify insertion of the *ivaAB* operon at the PP_5042 site. A colony with the correct 4090 bp insertion band was amplified again with oACB466 and oACB467, and the purified PCR product was sequenced with oACB468, oACB469, and oACB470 to verify the genomic sequence.

Initial Compositions of Bacterial Growth Media

Supplementary Table 21. Initial compositions of bacterial growth media (M9 minimal medium) with 10% v/v oxidized, methylated RCF oligomers. Preps A-C indicate separate replicates of the oxidation reaction. Note that acetic acid (acetate) is natively catabolized by *P. putida*.[27]

Compound	Concentration in M9 medium (mM)		
	**ox. RCF Prep A**	**ox. RCF Prep B**	**ox. RCF Prep C**
Protocatechuic Acid	0.00	0.00	0.00
Catechol	0.00	0.00	0.00
Total Muconic Acid (*cis,cis* + *cis,trans*)	0.00	0.00	0.00
Vanillic Acid	0.00	0.00	0.00
Isovanillic Acid	0.00	0.00	0.00
Vanillin	0.00	0.00	0.00
Veratric Acid	0.32	0.35	0.32
Veratraldehyde	0.05	0.05	0.15
Acetic acid	3.19	2.07	3.11

Numerical data for metabolites measured during cultivation of ACB263

Supplementary Table 22. Numerical data for metabolites measured during cultivation of ACB263. "Av" indicates the average of three biological replicates; "sd" indicates the standard deviation from the mean of those three replicates. All metabolite values are in mM. Cultures were run for 72 h, but no significant concentration changes were observed after 48 h, so the plots in **Supplementary Fig. 36** and **Supplementary Fig. 6D** were truncated for visual clarity.

ACB263 - M9 + 10% v/v oxidation products from methylated pine RCF, prep A														
**Time (h)**	**PCA_av**	**PCA_sd**	**COH_av**	**COH_sd**	**MA_av**	**MA_sd**	**VA_av**	**VA_sd**	**IVA_av**	**IVA_sd**	**VerA_av**	**VerA_SD**	**VAL_av**	**VAL_sd**
0	0		0		0		0		0		0.323357		0.048865	
7.333333	0	0	0	0	0.30825	0.055669	0	0	0	0	0.206861	0.034385	0	0
17	0	0	0	0	0.426554	0.137666	0	0	0	0	0.073314	0.000856	0	0
24	0	0	0	0	0.483792	0.115061	0	0	0	0	0	0	0	0
48	0	0	0	0	0.56126	0.085741	0	0	0	0	0	0	0	0
77	0	0	0	0	0.522154	0.111737	0	0	0	0	0	0	0	0

ACB263 - M9 + 10% v/v oxidation products from methylated pine RCF, prep B														
**Time (h)**	**PCA_av**	**PCA_sd**	**COH_av**	**COH_sd**	**MA_av**	**MA_sd**	**VA_av**	**VA_sd**	**IVA_av**	**IVA_sd**	**VerA_av**	**VerA_SD**	**VAL_av**	**VAL_sd**
0	0		0		0		0		0		0.352082		0.051453	
7.333333	0	0	0	0	0.254223	0.013667	0	0	0	0	0.169637	0.019334	0	0
17	0	0	0	0	0.364755	0.010477	0	0	0	0	0.066396	0.002562	0	0
24	0	0	0	0	0.390266	0.02783	0	0	0	0	0	0	0	0
48	0	0	0	0	0.451297	0.014736	0	0	0	0	0	0	0	0
77	0	0	0	0	0.415933	0.004407	0	0	0	0	0	0	0	0

ACB263 - M9 + 10% v/v oxidation products from methylated pine RCF, prep C														
**Time (h)**	**PCA_av**	**PCA_sd**	**COH_av**	**COH_sd**	**MA_av**	**MA_sd**	**VA_av**	**VA_sd**	**IVA_av**	**IVA_sd**	**VerA_av**	**VerA_SD**	**VAL_av**	**VAL_sd**
0	0		0		0		0		0		0.315258		0.148901	
7.333333	0	0	0	0	0.280631	0.001121	0	0	0	0	0.165039	0.004223	0	0
17	0	0	0	0	0.442303	0.015681	0	0	0	0	0.069863	0.002333	0	0
24	0	0	0	0	0.464399	0.010916	0	0	0	0	0	0	0	0
48	0	0	0	0	0.506234	0.014715	0	0	0	0	0	0	0	0
77	0	0	0	0	0.445537	0.017131	0	0	0	0	0	0	0	0

ACB263 - M9 + 2 mM veratrate														
**Time (h)**	**PCA_av**	**PCA_sd**	**COH_av**	**COH_sd**	**MA_av**	**MA_sd**	**VA_av**	**VA_sd**	**IVA_av**	**IVA_sd**	**VerA_av**	**VerA_SD**	**VAL_av**	**VAL_sd**
0	0		0		0		0		0		2.03782		0	
7.333333	0	0	0	0	0.858698	0.026715	0	0	0	0	0.947359	0.037175	0.003634	0.006295
17	0	0	0	0	1.229559	0.026298	0.043093	0.03733	0	0	0.511002	0.01399	0	0
24	0	0	0	0	1.263045	0.022249	0.074875	0.005568	0	0	0.498407	0.016122	0	0
48	0	0	0	0	1.842252	0.015091	0	0	0	0	0	0	0	0
77	0	0	0	0	1.831132	0.031438	0	0	0	0	0	0	0	0

ACB263 - M9 + 2 mM veratraldehyde														
**Time (h)**	**PCA_av**	**PCA_sd**	**COH_av**	**COH_sd**	**MA_av**	**MA_sd**	**VA_av**	**VA_sd**	**IVA_av**	**IVA_sd**	**VerA_av**	**VerA_SD**	**VAL_av**	**VAL_sd**
0	0		0		0		0		0		0		1.97381	
7.333333	0.143014	0.002897	0	0	0.897016	0.004444	0.012645	0.021902	0	0	0.541269	0.01194	0.206371	0.018999
17	0	0	0	0	1.629896	0.015409	0	0	0	0	0.214466	0.00628	0	0
24	0	0	0	0	1.669817	0.01911	0	0	0	0	0.189194	0.00202	0	0
48	0	0	0	0	1.886701	0.038725	0	0	0	0	0	0	0	0
77	0	0	0	0	1.892691	0.030842	0	0	0	0	0	0	0	0

Bioconversion Conversion

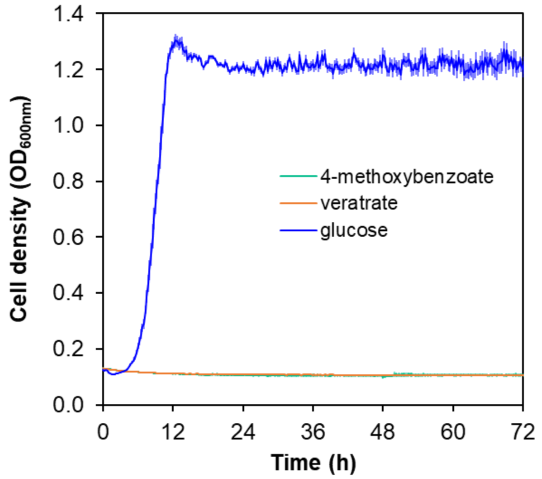

Supplementary Fig. 36. *P. putida* KT2440 wild-type does not utilize the *p*-methoxylated substrates 4-methoxybenzoate or veratrate. Biological triplicate overnight cultures of *P. putida* KT2440 wild-type were inoculated into wells of a microtiter plate filled with M9 minimal medium supplemented with 5 mM 4-methoxybenzoate (teal), 5 mM veratrate (orange), or 20 mM glucose (blue). The plate was incubated at 30 °C and 225 rpm with maximum shaking in a BioscreenC Pro (Growth Curves Ltd.), and the optical density at 600 nm was measured every 15 min. Error bars indicate the standard deviation from the mean of three biological replicates.

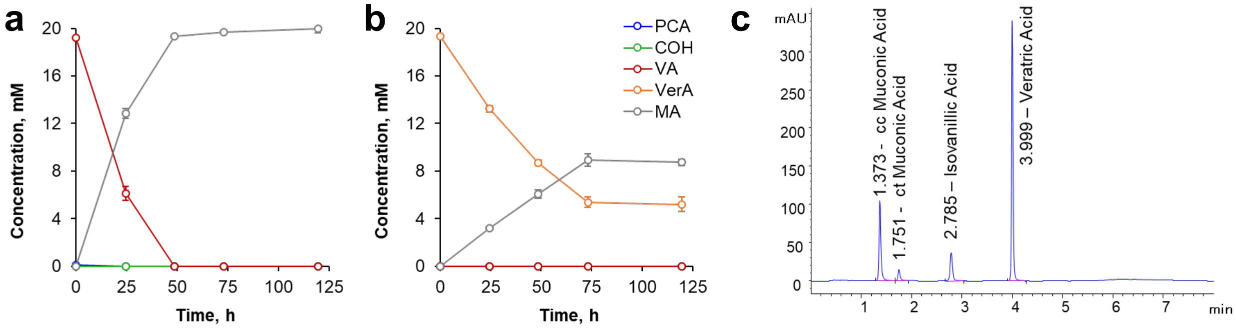

Supplementary Fig. 37. In trial experiments with *P. putida* strain ACB180, **(a)** the strain fully converted 20 mM vanillate to muconate. **(b)** However, conversion of 20 mM veratrate was incomplete and halted after ~75 h. **(c)** HPLC traces revealed that the incomplete conversion of veratrate was due to undesirable accumulation of isovanillate, likely the result of veratrate *O*-demethylation by VanAB. Error bars represent the standard deviation from the mean of three biological replicates. Metabolite abbreviations: PCA, protocatechuate; COH, catechol; VA, vanillate; VerA, veratrate; MA, muconate.

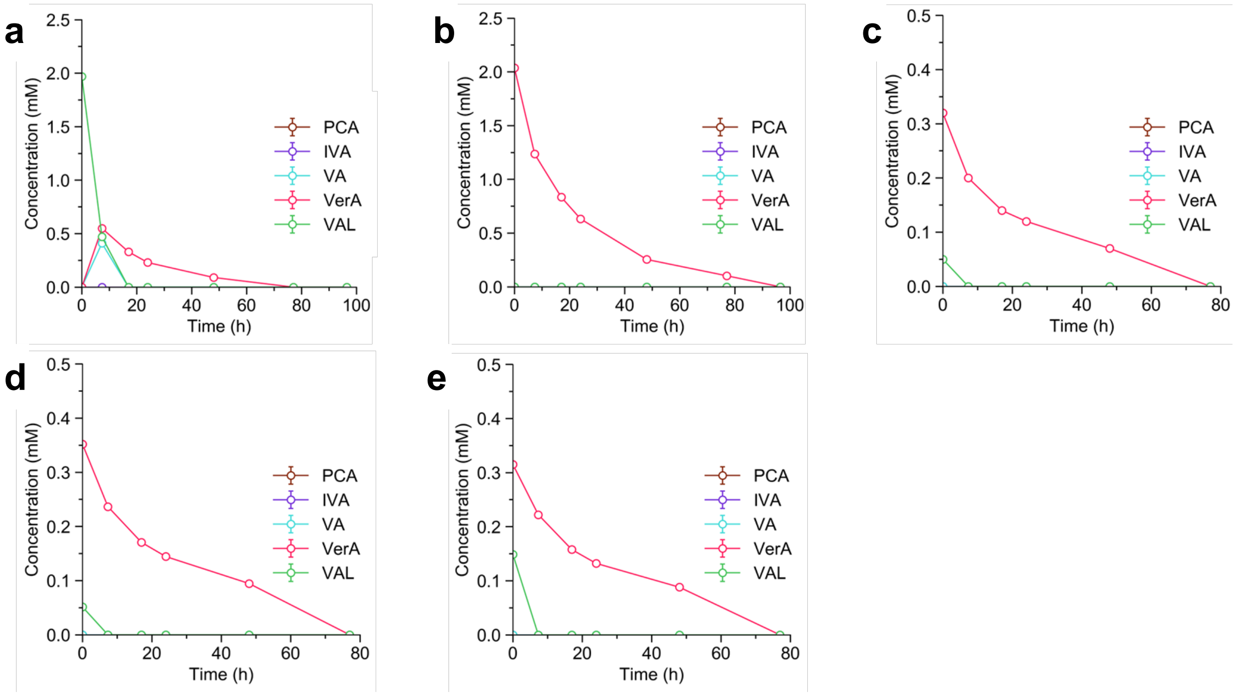

Supplementary Fig. 38. Metabolite quantification for cultivations of **(a)** *P. putida* strain ACB262 in M9 with 2 mM veratraldehyde, **(b)** strain ACB262 in M9 with 2 mM veratrate, and **(c-e)** strain ACB262 in M9 with 10% v/v oxidized RCF oligomers (each plot represents cultivation with a different replicate of the oxidation reaction). Cultures were conducted as singlets. Abbreviations: PCA, protocatechuate; IVA, isovanillate; VA, vanillate; VerA, veratrate; VAL, veratraldehyde.

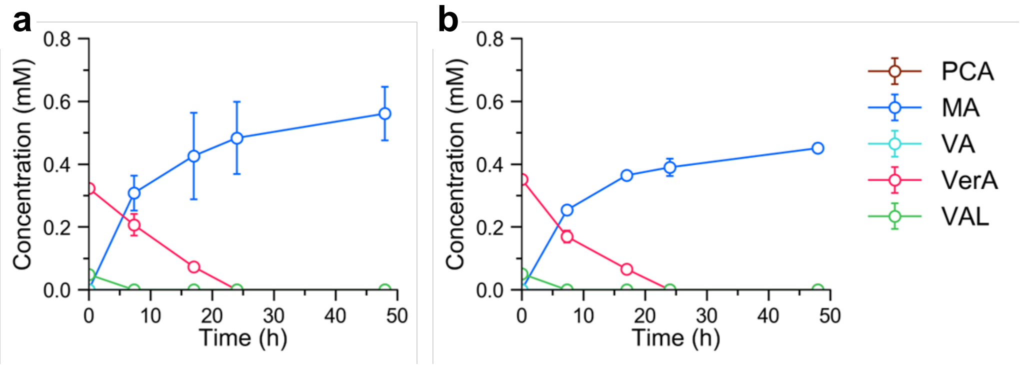

Supplementary Fig. 39. Strain ACB263 produces muconate from veratrate and veratraldehyde when grown in M9 + 10% v/v oxidation products from methylated pine RCF. Each Supplementary Fig. **(a)** and **(b)** represents one pine oligomers oxidation reaction under the standard conditions. Each data point represents an average value and standard deviation from three biological replicates. Abbreviations: PCA, protocatechuate; MA, muconate; VA, vanillate; VerA, veratrate; VAL, veratraldehyde.

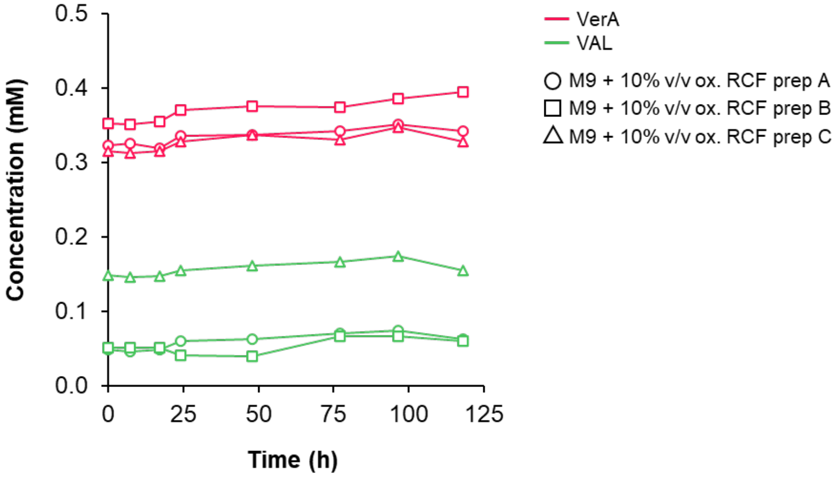

Supplementary Fig. 40. Cell-free M9 media containing 10% v/v of oxidation products from methylated RCF oligomers were incubated under the same conditions as the shaken flasks in **Supplementary Fig. 37** and **Supplementary Fig. 38**. No substantial changes were observed in the concentration of major monomer constituents, veratrate (VerA) and veratraldehyde (VAL).

Supplementary References

1. Jung J-C, Jung Y-J, Park O-S. A convenient one-pot synthesis of 4-hydroxycoumarin, 4-hydroxythiocoumarin, and 4-hydroxyquinolin-2 (1 H)-one. *Synth. Commun.* **31**, 1195-1200 (2001).

2. Curti C*, et al.* Streamlined, asymmetric synthesis of 8,4'-oxyneolignans. *J. Org. Chem.* **71**, 8552-8558 (2006).

3. Antonietta Dettori M*, et al.* 4-Substituted-2-methoxyphenol: suitable building block to prepare new bioactive natural-like hydroxylated biphenyls. *Lett. Drug Des. Discov.* **12**, 131-139 (2015).

4. Llevot A, Grau E, Carlotti S, Grelier S, Cramail H. ADMET polymerization of bio-based biphenyl compounds. *Polym. Chem.* **6**, 7693-7700 (2015).

5. Constantin M-A, Conrad J, Beifuss U. Laccase-catalyzed oxidative phenolic coupling of vanillidene derivatives. *Green Chem.* **14**, 2375-2379 (2012).

6. Grelier S, Cramail H, Llevot A, Carlotti S, Grau E. Process for preparing biphenyl compounds. U.S. Patent 16/150,925 (2019).

7. Liang YF, Wu K, Song S, Li X, Huang X, Jiao N. I2- or NBS-catalyzed highly efficient alpha-hydroxylation of ketones with dimethyl sulfoxide. *Org. Lett.* **17**, 876-879 (2015).

8. Sahoo MK, Mhaske SB, Argade NP. Facile routes to alkoxymaleimides/maleic anhydrides. *Synthesis* **2003**, 0346-0349 (2003).

9. Enomoto Y, Iwata T. Synthesis of biphenyl polyesters derived from divanillic acid, and their thermal and mechanical properties. *Polymer* **193**, 122330 (2020).

10. Brewer CP, Cooke LM, Hibbert H. Studies on lignin and related compounds. LXXXIV. the high pressure hydrogenation of maple wood: hydrol lignin1. *J. Am. Chem. Soc.* **70**, 57-59 (1948).

11. Sluiter A*, et al.* Determination of structural carbohydrates and lignin in biomass. NREL Technical Report (2008).

12. Sluiter JB, Ruiz RO, Scarlata CJ, Sluiter AD, Templeton DW. Compositional analysis of lignocellulosic feedstocks. 1. Review and description of methods. *J. Agric. Food Chem.* **58**, 9043-9053 (2010).

13. Gu NX*, et al.* Autoxidation catalysis for carbon–carbon bond cleavage in lignin. *ACS Cent. Sci. In Press*, (2023).

14. Zhang J*, et al.* Synthesis, antiepileptic effects, and structure-activity relationships of alpha-asarone derivatives: In vitro and in vivo neuroprotective effect of selected derivatives. *Bioorg. Chem.* **115**, 105179 (2021).

15. Hitce J, Crutizat M, Bourdon C, Vivès A, Marat X, Dalko-Csiba M. Flash-metathesis for the coupling of sustainable (poly) hydroxyl β-methylstyrenes from essential oils. *Green Chem.* **17**, 3756-3761 (2015).

16. Mori N, Furuta A, Watanabe H. Electochemical asymmetric dimerization of cinnamic acid derivatives and application to the enantioselective syntheses of furofuran lignans. *Tetrahedron* **72**, 8393-8399 (2016).

17. Yue F, Lu F, Regner M, Sun R, Ralph J. Lignin-derived thioacidolysis dimers: reevaluation, new products, authentication, and quantification. *ChemSusChem* **10**, 830-835 (2017).

18. Gala D, Puar MS, Das PR, Kugelman M, Dibenedetto DJ. Decomposition of α-hydroxyaryl ketones and characterization of some unusual products. *J. Pharm. Sci.* **81**, 1199-1203 (1992).

19. Endo A, Kitahara N, Oka H, Miguchi-Fukazawa Y, Terahara A. Isolation of 4.5-dihydroxyisophthalic acid an inhibitor of brain glutamate decarboxylase, produced by a Streptomyces species. *Eur. J. Biochem.* **82**, 257-259 (1978).

20. Kapat A, Sperger T, Guven S, Schoenebeck F. E-Olefins through intramolecular radical relocation. *Science* **363**, 391-396 (2019).

21. Jang JH*, et al.* Multi-pass flow-through reductive catalytic fractionation. *Joule*, (2022).

22. Choi K-H, Kumar A, Schweizer HP. A 10-min method for preparation of highly electrocompetent *Pseudomonas aeruginosa* cells: application for DNA fragment transfer between chromosomes and plasmid transformation. *J. Microbiol. Methods* **64**, 391-397 (2006).

23. Johnson CW, Beckham GT. Aromatic catabolic pathway selection for optimal production of pyruvate and lactate from lignin. *Metab. Eng.* **28**, 240-247 (2015).

24. Lynch MD, Gill RT. Broad host range vectors for stable genomic library construction. *Biotechnol. Bioeng.* **94**, 151-158 (2006).

25. Jayakody LN*, et al.* Thermochemical wastewater valorization via enhanced microbial toxicity tolerance. *Energy & Environ. Sci.* **11**, 1625-1638 (2018).

26. Kuatsjah E*, et al.* Debottlenecking 4-hydroxybenzoate hydroxylation in *Pseudomonas putida* KT2440 improves muconate productivity from p-coumarate. *Metab. Eng.* **70**, 31-42 (2022).

27. Yang S, Li S, Jia X. Production of medium chain length polyhydroxyalkanoate from acetate by engineered Pseudomonas putida KT2440. *J. Ind. Microbiol. Biotechnol.* **46**, 793-800 (2019).
